# Galectin-1 confers resistance to doxorubicin in hepatocellular carcinoma cells through modulation of P-glycoprotein expression

**DOI:** 10.1038/s41419-022-04520-6

**Published:** 2022-01-24

**Authors:** Pablo Carabias, María V. Espelt, María L. Bacigalupo, Paola Rojas, Luciana Sarrias, Ayelén Rubin, Nicolás A. Saffioti, María T. Elola, Juan P. Rossi, Carlota Wolfenstein-Todel, Gabriel A. Rabinovich, María F. Troncoso

**Affiliations:** 1 Universidad de Buenos Aires, Consejo Nacional de lnvestigaciones Científicas y Técnicas, Instituto de Química y Fisicoquímica Biológicas, Departamento de Química Biológica, Facultad de Farmacia y Bioquímica, Buenos Aires, Argentina; 2 Laboratorio de Carcinogénesis Hormonal, Instituto de Biología y Medicina Experimental, Consejo Nacional de lnvestigaciones Científicas y Técnicas, Buenos Aires, Argentina; 3 Laboratorio de Glicomedicina, Instituto de Biología y Medicina Experimental, Consejo Nacional de lnvestigaciones Científicas y Técnicas, Buenos Aires, Argentina; 4 Facultad de Ciencias Exactas y Naturales, Universidad de Buenos Aires, Buenos Aires, Argentina

**Keywords:** Glycobiology, Liver cancer

## Abstract

Galectin-1 (GAL1), a β-galactoside-binding protein abundantly expressed in the tumor microenvironment, has emerged as a key mechanism of chemoresistance developed by different tumors. Although increased expression of GAL1 is a hallmark of hepatocellular carcinoma (HCC) progression, aggressiveness and metastasis, limited information is available on the role of this endogenous lectin in HCC resistance to chemotherapy. Moreover, the precise mechanisms underlying this effect are uncertain. HCC has evolved different mechanisms of resistance to chemotherapy including those involving the P-glycoprotein (P-gp), an ATP-dependent drug efflux pump, which controls intracellular drug concentration. Here, we investigated the molecular mechanism underlying GAL1-mediated chemoresistance in HCC cells, particularly the involvement of P-gp in this effect. Our results show that GAL1 protected HepG2 cells from doxorubicin (DOX)- and sorafenib-induced cell death in vitro. Accordingly, GAL1-overexpressing HepG2 cells generated DOX-resistant tumors in vivo. High expression of GAL1 in HepG2 cells reduced intracellular accumulation of DOX likely by increasing P-gp protein expression rather than altering its membrane localization. GAL1-mediated increase of P-gp expression involved activation of the phosphatidylinositol-3 kinase (PI3K) signaling pathway. Moreover, ‘loss-of-function’ experiments revealed that P-gp mediates GAL1-driven resistance to DOX, but not to sorafenib, in HepG2 cells. Conversely, in PLC/PRF/5 cells, P-gp protein expression was undetectable and GAL1 did not control resistance to DOX or sorafenib, supporting the critical role of P-gp in mediating GAL1 effects. Collectively, our findings suggest that GAL1 confers chemoresistance in HCC through mechanisms involving modulation of P-gp, thus emphasizing the role of this lectin as a potential therapeutic target in HCC.

## Introduction

Hepatocellular carcinoma (HCC), the most common type of liver cancer, has a poor prognosis accounting for the second leading cause of cancer-related deaths [1, 2]. It usually develops from liver fibrosis and cirrhosis irrespective of the etiology of the liver disease (chronic hepatitis B or C virus infection, excessive alcohol consumption, nonalcoholic steatohepatitis) [3]. Regarding treatment, when HCC is diagnosed at early stages, partial hepatectomy is the therapeutic choice; however, in most patients tumors are not detected early and progress. In intermediate stages, transarterial chemoembolization with doxorubicin (DOX), a topoisomerase II inhibitor that induces apoptosis, is the treatment of choice [4]. During the past decade, targeted molecular therapy with sorafenib, a multikinase inhibitor with antiproliferative and antiangiogenic properties has slightly improved survival in patients with advanced-stage HCC. Moreover, immunotherapy modalities are being explored in combination with targeted molecular therapy for treatment of these tumors. Nevertheless, HCC still has a high mortality rate, largely because of high recurrence rate, metastasis, and resistance to chemotherapy [5, 6].

A diverse range of molecular mechanisms have been implicated in drug resistance [7, 8]. ATP-binding cassette (ABC) transporter family includes ATP-dependent pumps that cause the efflux of hydrophobic compounds and xenobiotics such as chemotherapeutic drugs. The most studied protein involved in multidrug resistance (MDR) is P-glycoprotein (P-gp; *ABCB1/MDR1*). P-gp is overexpressed in many tumors (causing intrinsic MDR) and its expression can be induced by chemotherapy causing acquired MDR [9]. In HCC, P-gp overexpression is associated with chemotherapy failure [10, 11]. In addition, prolonged treatment with chemotherapeutic drugs can induce an increase in P-gp expression in HCC cells [12].

Galectin-1 (GAL1) is a glycan-binding protein with affinity for β-galactoside-containing glycoconjugates. This lectin exerts key roles in many physiological and pathological processes [13, 14]. Within the tumor microenvironment, GAL1 plays important roles in modulation of cell adhesion, tumor transformation, growth, invasion and metastasis, angiogenesis, and tumor-immune escape [15–17]. In normal adult liver, GAL1 levels are undetectable [18], whereas in HCC this lectin is overexpressed due to increased mRNA levels generated by hypo-methylation of the *LGALS1* gene promoter [19]. GAL1 overexpression in HCC correlates with tumor aggressiveness, metastasis, and enhanced risk of postpartial-hepatectomy recurrence [20, 21]. Thus, GAL1 has emerged as a potential biomarker of HCC poor prognosis and a therapeutic target of this malignant disease [22].

Previously, we described that GAL1 promotes HCC cell adhesion and polarization favoring tumor growth and metastasis in vivo [23]. Notably, at early stages of chronic liver pathology GAL1 may act as a protective anti-inflammatory agent, whereas at late stages of the disease it may display pro-tumorigenic roles [24, 25]. Recently, we found that growth hormone upregulates GAL1 expression in mouse liver, suggesting that this lectin could also be implicated in hormone-driven liver carcinogenesis [26]. Furthermore, we demonstrated that GAL1 overexpression induces epithelial-mesenchymal transition (EMT) in HepG2 HCC cells [27], a key process that contributes to cancer cell dissemination and confers drug resistance [28, 29]. Besides, our results demonstrated that secretion of GAL1 by HCC cells induced liver sinusoidal endothelial cell (SEC) proliferation and migration. Moreover, GAL1 promoted glycan-dependent HCC cell adhesion to SECs. Also, we identified GAL1 as a modulator of HepG2 cell proliferation and sensitivity to transforming growth factor β_1_ (TGF-β_1_)-induced growth inhibition [30].

Interestingly, recent studies have shed light on the role of GAL1 in HCC chemoresistance. Increased GAL1 expression in HCC patients’ sera or tumor tissue was associated with low clinical efficacy of sorafenib treatment and poor survival outcome [31, 32]. In addition, GAL1-induced autophagy has been proposed as a possible mechanism involved in HCC resistance to cisplatin, another chemotherapeutic drug [33]. However, in spite of considerable progress, the precise mechanisms underlying this effect remain unclear. Thus, we undertook this study to investigate the molecular basis of GAL1-mediated chemoresistance in HCC cells.

## Materials and methods

### Reagents

Bovine serum albumin, aprotinin, phenylmethylsulfonyl fluoride (PMSF), poly-L-lysine, PD98059, wortmannin, G418 disulfate salt, puromycindihydrochloride, doxorubicin hydrochloride (DOX), probenecid, verapamil, 3-(4,5-Dimethyl-2-thiazolyl)-2,5-diphenyl-2H-tetrazolium bromide (MTT), H-89 dihydrocloride hydrate, bisBenzimide H33258 (Hoescht), 1,4-diazabicyclo[2.2.2] octane (DABCO), 4′,6′diamidino-2-phenylindole (DAPI), tetramethyl-rhodamineisothiocyanate (TRITC)-phalloidin, and horseradish peroxidase (HRP)-conjugated anti-rabbit IgG were purchased from Merck KGaA (Darmstadt, Germany). Dulbecco’s modified Eagle medium (DMEM), L-glutamine, and trypsin/EDTA solution from GIBCO® and 5-chloromethylfluorescein diacetate (CMFDA) from Molecular Probes® were purchased from Thermo Fisher Scientific Inc. (Waltham, MA, USA). Bovine fetal serum was from Natocor (Córdoba, Argentina). Anti-GAL1 antibody (sc-19277), anti-P-gp antibody (MDR1 (D-11): sc-55510), anti-β-tubulin antibody, horseradish peroxidase (HRP)-conjugated anti-mouse IgG and sorafenib were from Santa Cruz Biotechnology, Inc. (Santa Cruz, CA, USA). Anti-multidrug resistance-associated protein 2 (MRP2) monoclonal antibody (M2III-6) was obtained from ALEXIS® Biochemicals-Enzo Life Sciences, Inc. (Farmingdale, NY, USA). Fluorescein isothiocyanate (FITC)-conjugated anti-IgG antibody was purchased from Jackson ImmunoResearch Laboratories (West Grove, PA, USA). Pegylated liposomal DOX hydrochloride, a formulation of DOX in polyethylene glycol liposomes developed to decrease the risk of cardiotoxicity experienced with conventional DOX while preserving the anti-tumor efficacy (DOXPLAX, 20 mg/10 ml), was obtained from LKM (Buenos Aires, Argentina).

### Cell culture and transfections

The human HCC cell lines HepG2/C3A (CRL-10741, a clonal derivative of HepG2 cell line HB-8065, ATCC, Manassas, VA, USA) and PLC/PRF/5 (CRL-8024, ATCC) were cultured in DMEM, 10% v/v serum, 2 mM L-glutamine and antibiotics in a humidified atmosphere of 5% CO_2_ at 37 °C. All the experiments were performed using cells of passage number less than 30 and maintained in a logarithmic growth phase. Cells were tested for mycoplasma contamination. Transfections to overexpress GAL1 were performed as previously described [27, 30]. Briefly, cells were transfected with pcDNA3.1-*LGALS1* [34] or pcDNA3.1 expression vector (Thermo Fisher Scientific Inc.) alone as control, using Lipofectamine 2000 (Thermo Fisher Scientific Inc.). Stable GAL1-overexpressing cells were selected by G418 resistance. Transfections to knock down GAL1 were performed with Galectin-1 shRNA Plasmid (h) (sc-35441-SH, Santa Cruz Biotechnology, Inc.), a pool of three target-specific lentiviral vector plasmids each encoding 19–25 nt (plus hairpin) shRNAs designed to knock down gene expression. Control shRNA plasmid-A (sc-108060, Santa Cruz Biotechnology, Inc.) encodes a scrambled shRNA sequence that does not lead to the specific degradation of any known cellular mRNA, and was used as a negative control. After transfection, cells stably expressing shRNA were selected by puromycin resistance. P-gp silencing experiments were performed with MDR1 siRNA (h) (sc-29395, Santa Cruz Biotechnology, Inc.), a target-specific 19–25 nt siRNA. Control siRNA-A (sc-37007, Santa Cruz Biotechnology, Inc.), a non-targeting scrambled siRNA, was used as a negative control. Overexpression and silencing efficiencies were assessed by western blot.

### Western blot analysis

Cells were homogenized in lysis buffer (100 mM Tris, pH 7.4, 1% v/v Triton X-100, 10 mM EDTA, 2 mM PMSF and 0.012–0.034 IU/ml aprotinin). After 45 min, cell lysates were centrifuged for 10 min at 14,000 × *g* at 4 °C and supernatants were collected. Twenty-five micrograms of protein per sample in sample buffer (125 mM Tris-HCl, pH 6.8; 4% w/v SDS; 20% v/v glycerol, 10% v/v β-mercaptoethanol; 0.005% w/v bromophenol blue) were heated at 100 °C for 5 min. To evaluate P-gp expression, samples were heated at 40 °C for 30 min. To analyze the involvement of phosphatidylinositol 3-kinase (PI3K), ERK1/2 mitogen-activated protein kinase (MAPK) kinase (MEK) and protein kinase A (PKA) signaling pathways in P-gp expression modulated by GAL1, cells were incubated for 24 h in the presence of the PI3K inhibitor wortmannin (10 µM), MAPK inhibitor PD98059 (25 µM) in dimethyl sulfoxide (DMSO) or PKA inhibitor H89 (10 µM), and then homogenized as previously described. Proteins were separated by SDS-PAGE, transferred to polyvinylidene-difluoride (PVDF) membranes (Bio-Rad Laboratories, Inc., Hercules, CA, USA) and immunoblotted. Bands were detected by chemiluminescence (Amersham ECL prime Western Blotting Detection Reagents, GE Healthcare, Uppsala, Sweden). Densitometric analysis of protein levels was performed using ImageJ software (U.S. National Institutes of Health, Bethesda, MD, USA; http://rbsweb.nih.gov/ij/).

### Cell viability and apoptosis assays

To evaluate cell viability, cells were cultured in 96-well plates at a density of 15,000 cells per well with DMEM plus 10% serum for 24 h. Then, cells were washed with PBS and incubated with increasing concentrations of DOX for 48 or 72 h or sorafenib for 24 h. To evaluate P-gp involvement in GAL1-mediated drug resistance, cells were cultured for 24 h with serum, washed with PBS and pre-incubated with increasing concentrations of P-gp inhibitor, verapamil, or MRP2 inhibitor, probenecid, for 30 min. Then, culture media were replaced, cells were incubated for another 24 h with increasing concentrations of inhibitors and DOX (2 µM) and cell viability was evaluated. To further confirm the specific contribution of P-gp, cells were cultured in 96-well plates at a density of 7500 cells per well with DMEM plus 10% serum for 24 h, and transfected with P-gp specific siRNA or non-targeting scrambled siRNA, as described before (see the section “Cell culture and transfections”). At 48 h post-transfection, cells were incubated with DOX (2 µM) or sorafenib (30 µM) and cell viability was determined after another 24 h. Cell viability was evaluated by the MTT colorimetric assay as described before [35] at the indicated times, and the results were expressed as percentage of cell viability. In some assays, the experimental data were fitted to dose–response curves by non-linear regression using the statistical program GraphPad Prism for Windows, version 6.01.

To determine the percentage of apoptosis, HepG2 cells were cultured in 24-well plates on sterilized 12-mm-diameter glass coverslips coated with 0.1% w/v poly-L-lysine at a density of 50,000 cells per coverslip (30–40% confluence) for 24 h. Then, cells were washed with PBS and incubated with 5 µM DOX for 24 or 48 h. Cells were fixed with methanol at −20 °C for 10 min. Nuclei were stained with 50 ng/ml of Hoechst 33258 for 10 min. Coverslips were mounted with DABCO on glass slides and photographed on a Nikon TE-200 epifluorescence-inverted microscope (Tokyo, Japan). Nuclear morphology was analyzed from, at least, 20 cells/field, 20 fields/coverslip, and 2 coverslips per condition. The percentage of apoptotic nuclei (with condensed chromatin) was calculated as the number of altered nuclei × 100/total number of nuclei, using ImageJ software.

### Chemoresistance in vivo

NOD/LySz-scid/IL-2Rgamma null (NSG) mice were purchased from the Jackson Laboratories (Bar Harbor, ME USA) and bred at the Instituto de Biología y Medicina Experimental (IBYME), Consejo Nacional de lnvestigaciones Científicas y Técnicas. NSG mice have impaired innate and acquired immunity as they lack mature T cells, B cells, and natural killer (NK) cells and were used for xenotransplantation of human HCC cell line HepG2. All studies were performed according to protocols approved by the Instituto de Biología y Medicina Experimental-Instructional Animal Care and Use Committee (IACUC) committee and in agreement with the National Institutes of Health (NIH) Guide for Care and Use of Laboratory Animals (National Research Council, 2011). Male mice (*n* = 12) of adult age (2 months) fed ad libitum housed in ventilated boxes, between 3 or 4 animals per box, were used in rooms with regulated temperature (20 ± 2 °C), with a 12 h light-dark cycle and in pathogen-free conditions. All the used materials, boxes, beds, food, and water were sterilized by autoclaving. HepG2-M or HepG2-GAL1 cells (5 × 10^6^/100 µl PBS) were subcutaneously inoculated into each anesthetized (100 mg/ml ketamine, 10 mg/ml xylazine) animal. Thus, two groups of mice were randomized defined, each one composed of 6 animals. Subcutaneous tumor growth was measured every 3–4 day with a Vernier caliper throughout the experiment. Tumor volume was determined applying the following formula: ${\it{V}} = {\it{a}}^2\frac{l}{2}$, *V* is the calculated tumor volume; *a* and *l* are manually measured tumor width and length, respectively. When the tumors reached ~0.06 cm^3^, mice were assigned (3 animals per group) to control (saline solution) or DOX treatment (pegylated liposomal DOX hydrochloride, 4.5 mg/kg), and intravenously (i.v.) treated once a week for 3 weeks. A 9 mg/kg dose of pegylated DOX was shown to induce complete tumor regression and mild side effects in mice; therefore we selected a lower dose that was still effective [36]. At the end of the experiment, mice were sacrificed, and tumor samples were collected for protein expression analysis by western blot. Tumor volume was calculated and plotted versus time to determine response to therapy. Blind analysis of animals/samples from treatment with respect to control groups was performed.

### Bile pseudocanaliculi secretory function and intracellular DOX accumulation

To study bile pseudocanaliculi secretory function, HepG2 cells expressing different levels of GAL1 were cultured in 24-well plates on sterilized 12-mm-diameter glass coverslips coated with 0.1% w/v poly-L-lysine at a density of 50,000 cells per coverslip (30–40% confluence) for 24 h. Then, each coverslip was mounted in a chamber placed on the stage of a Nikon TE-200 epifluorescence-inverted microscope (Tokyo, Japan). Living cells were incubated with 20 µM DOX (excitation wavelength: 470 nm, emission wavelength: 595 nm) for 1 h and one field was photographed. Cells were then loaded with 2.5 µM 5-chloromethylfluorescein diacetate (CMFDA) for 1 h and the same field previously focused was photographed. Hepatocytes capture and metabolize this compound, generating fluorescent glutathione methylfluorescein (GS-MF; excitation wavelength: 495 nm, emission wavelength: 510 nm), which is actively secreted to the canaliculi by MRP2 transporter [37]. Thus, GS-MF fluorescence was used to visualize canalicular structures and to analyze DOX secretion to pseudocanaliculi. Ten coverslips/cell type were analyzed.

The intracellular DOX accumulation was estimated by a fluorimetric method, as described by Lecureur et al. [38]. HepG2 cells expressing different levels of GAL1 were cultured in 35 mm plates for 24 h, and then exposed to DOX (20 µM) for 1, 2, 4, and 6 h. After extensive washing, cells were scraped and homogenized in lysis buffer (10 mM Tris-HCl, pH 7, 100 mM NaCl, 10 mM EDTA, 0.01% v/v Triton X-100). The amount of DOX uptake by the cells was determined with a Multi-Mode Microplate Reader Synergy™ HT (BioTek® Instruments, Inc., Winooski, VT, USA) (excitation filter: 485/20 nm, emission filter: 590/35 nm). To estimate DOX mass in cell homogenates, a calibration curve was performed. Fluorescence intensity as function of DOX had a linear behavior in the entire range of concentrations observed in this work. In parallel, protein content in cell lysate aliquots was determined. Therefore, intracellular DOX accumulated by each cell line at different times was expressed as DOX mass (pmol) per protein mass (µg).

### P-gp immunolocalization and bile pseudocanaliculi staining

HepG2 cells expressing different levels of GAL1 cultured on poly-L-lysine-coated coverslips for 24 h were fixed with 4% w/v formaldehyde for 15 min and permeabilized with PBS-0.5% v/v Triton X-100 for 10 min. Then, cells were incubated for 1 h in PBS-0.1% v/v Triton X-100 containing 1% w/v BSA to block nonspecific binding sites. Cells were incubated overnight at 4 °C with anti-P-gp (1:50). Coverslips were then incubated with corresponding FITC-conjugated anti-IgG antibody (1:100) for 1 h. To stain F-actin, which is accumulated underneath canalicular membranes, cells were treated with 2 µg/ml TRITC-phalloidin for 20 min. Cell nuclei were stained with 0.5 mg/ml DAPI. Coverslips were mounted with DABCO on glass slides, and cells were analyzed with an Olympus BX50 epifluorescence microscope (Olympus, Tokyo, Japan). Ten fields per coverslip were photographed.

### Statistical analysis

Data were analyzed using GraphPad Prism Software (GraphPad Software Inc, San Diego, CA, USA). Results are expressed as the mean ± standard error of the mean (SEM) from, at least, four independent experiments, unless indicated otherwise. Statistical analysis was done applying one- or two-way analysis of the variance (ANOVA) and Bonferroni post-test, or paired Student’s *t*-test. Data met the assumptions of the used tests. All tests were two-tailed, *p* values < 0.05 were considered to be significant.

## Results

### GAL1 protects HepG2 HCC cells from DOX- and sorafenib-induced cell death in vitro

**Fig. 1:**
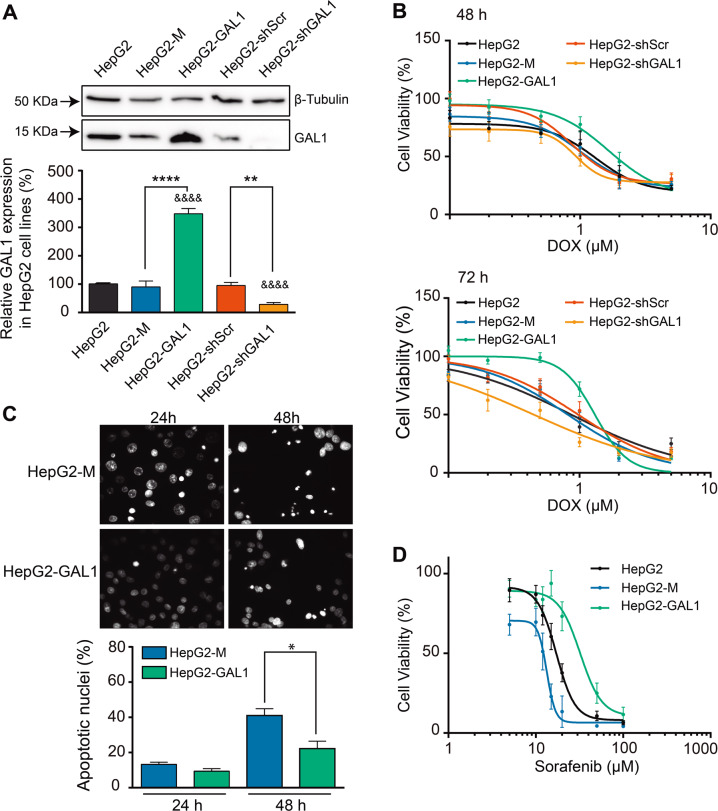
GAL1 protects HepG2 HCC cells from DOX- and sorafenib-induced cell death in vitro. **A** Western blot and densitometric analysis showing relative GAL1 expression in non-transfected (HepG2) and transfected with pcDNA3.1-*LGALS1* (HepG2-GAL1), expression vector without insert (HepG2-M), GAL1 shRNA plasmid (HepG2-shGAL1) or scrambled shRNA plasmid (HepG2-shScr) cell lysates. β-tubulin was used as loading control (*n* = 5). **p < 0.01, ****p < 0.0001, with respect to the corresponding controls. ^&&&&^*p* < 0.0001 with respect to HepG2 cells. **B** Cell viability (MTT assay) in GAL1-overexpressing, GAL1-silenced and control cells incubated with increasing concentrations of DOX (0-5 µM) for 48 or 72 h. Results are expressed as the mean of cell viability percentage with respect to the corresponding untreated cell line (100%) ± SEM (*n* = 8). The experimental data were fitted to dose–response curves by non-linear regression as described in “Materials and methods”. **C** Apoptosis in HepG2-M and HepG2-GAL1 cells after 5 µM DOX treatment for 24 or 48 h. Cells were stained with nuclear fluorescent dye Hoechst 33258 and nuclear morphology was analyzed by epifluorescence microscopy (40X). Cells that underwent apoptosis showed condensed and/or fragmented nuclei (upper panel). Percentages of apoptotic nuclei were calculated as described in *Materials and Methods*. Results are expressed as the mean ± SEM (*n* = 4) (lower panel). *p < 0.05 with respect to HepG2-M cells. **D** Cell viability (MTT assay) in GAL1-overexpressing and control HepG2 cells cultured with increasing concentrations of sorafenib (5–100 µM) for 24 h. Results are expressed as the mean of cell viability percentage relative to untreated cells (100%) ± SEM (*n* = 6). The experimental data were fitted to dose–response curves by non-linear regression.

To unravel the function of GAL1 in HCC cell drug resistance, we used the well-differentiated and low-invasive human HCC cell line, HepG2. GAL1-overexpressing cells (HepG2-GAL1) showed approximately 3.5-fold higher expression of GAL1, while GAL1-silenced cells (HepG2-shGAL1) exhibited a 70% decrease in GAL1 protein levels, as determined against the corresponding control cells (Fig. 1A).

To determine whether different levels of GAL1 expression affect viability in HepG2 cells exposed to chemotherapeutic drugs, cells were cultured in the presence of increasing concentrations of DOX for 48 or 72 h. Percentages of cell viability were assessed by the MTT assay and plotted versus DOX concentration on dose–response curves for each time. GAL1-overexpressing cells displayed viability curves shifted to the right with respect to HepG2-M control cells, whereas cells with decreased levels of GAL-1 showed curves shifted to the left with respect to HepG2-shScr control cells (Fig. 1B). By comparison of half-maximal inhibitory concentration (IC_50_) values, we observed that overexpression of GAL1 significantly protected HepG2 cells from DOX exposure while GAL1 silencing sensitized these cells to the cytotoxic effects of this drug (Table 1). After incubation of HepG2-GAL1 cells with DOX for 24 h, we also found an increase in cell viability with respect to HepG2-M-treated cells (65 ± 6% vs. 20 ± 4%, respectively, at 10 µM DOX); however in this case, IC_50_ value could not be determined. These results were confirmed using Hoechst staining and analysis of nuclear morphology by fluorescence microscopy. HepG2-GAL1 cells showed a significantly lower percentage of apoptotic nuclei than control cells when both were incubated with DOX (Fig. 1C).

**Table 1:** GAL1 protects HepG2 HCC cells from DOX-and sorafenib-induced cell death in vitro.

	DOX IC_50_ (µM) (CI_95_)		Sorafenib IC_50_ (µM) (CI_95_)
	48 h	72 h	24 h
HepG2	1.13 (0.77–1.67)	0.86 (0.67–1.12)	17.18 (15.00–19.66)
HepG2-M	1.19 (0.89–1.59)	0.81 (0.69–0.96)	13.36 (11.79–15.13)
HepG2-GAL1	1.97 (1.58–2.47) (165%*)	1.31 (1.17–1.46) (162%*)	32.25 (22.31–46.61) (241%*)
HepG2-shScr	1.33 (1.04–1.72)	0.97 (0.78–1.21)	nd
HepG2-shGAL1	0.84 (0.55–1.29) (63%^#^)	0.44 (0.33–0.6) (45%^#^)	nd

HepG2 cells with different levels of GAL1 expression were cultured in the presence of increasing concentrations of DOX (0–5 µM) or sorafenib (5–100 µM) for the indicated times. Percentages of cell viability were assessed by MTT assay and plotted versus DOX or sorafenib concentration on dose–response curves for each time. DOX or sorafenib half-maximal inhibitory concentration (IC_50_) values were obtained from dose–response viability curves for each cell line and incubation time (Fig. 1B, D) using the statistical program GraphPad Prism for Windows, version 6.01. *, ^#^, with respect to IC_50_ values obtained for HepG2-M and HepG2-shScr cells, respectively.

*CI*_*95*_ 95% confidence interval; *nd* not determined.

To confirm the role of GAL1 in HCC chemoresistance, we investigated whether overexpression of this lectin confers resistance to sorafenib in HepG2 cells. Remarkably, HepG2-GAL1 cells exhibited augmented viability compared with control cells incubated with sorafenib (Fig. 1D, Table 1).

These results demonstrate the involvement of GAL1 in HepG2 HCC cell resistance to death induced by both DOX and sorafenib.

### GAL1 overexpression induces resistance to DOX in HepG2-derived tumors in vivo

**Fig. 2:**
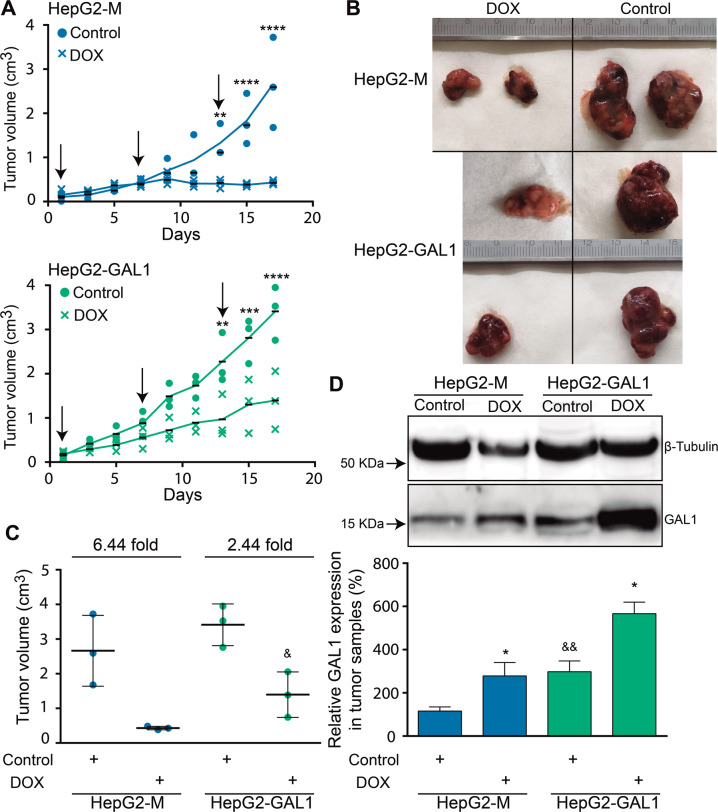
Overexpression of GAL-1 induces resistance to DOX in HepG2-derived tumors in vivo. **A** HepG2-M (upper panel) or HepG2-GAL1 (lower panel) (5 × 10^6^) cells were subcutaneously inoculated into immunodeficient NSG (NOD scid gamma) mice. When the tumors reached approximately 0.06 cm^3^ (treatment start time), animals were treated with saline solution (control) intravenously (i.v.) or DOX (pegylated liposomal doxorubicin hydrochloride, 4.5 mg/kg i.v.). Treatment days are indicated with arrows. Tumor volume was measured as described in “Materials and methods” (3 animals per group) every 3–4 days along the experiment. **p < 0.01, ***p < 0.001, ****p < 0.0001 comparing control vs DOX-treated animals for each cell line. At the end of the experiment, mice were euthanized and tumors removed. **B** Photographs of representative tumors are shown. **C** Response to therapy was evaluated by the mean ± standard deviation (SD) of tumor volume at the end of the experiment. ^&^*p* < 0.05 with respect to DOX HepG2-M-derived tumors. Results shown in **A**–**C** are representative of two independent experiments. **D** Western blot and densitometric analysis showing relative GAL-1 expression in tumor samples. β-tubulin was used as loading control (*n* = 3). *p < 0.05 compared with tumors from control mice inoculated with the corresponding cell line. ^&&^*p* < 0.01 with respect to control HepG2-M-derived tumors.

In previous studies, we found that GAL1 overexpression in HepG2 cells enhances tumor growth in immunodeficient mice [23]. To investigate the involvement of GAL1 in HCC chemoresistance in vivo, here we evaluated whether overexpression of this lectin in HepG2 cells could generate DOX-resistant tumors. We inoculated HepG2-M or HepG2-GAL1 cells into immunodeficient NSG mice. Treatment with DOX started when HepG2-M and HepG2-GAL1 cells generated palpable tumors. Although control mice from both HepG2-M and HepG2-GAL1 cells developed larger tumors than DOX-treated animals across the whole experiment, we observed a significant decrease in the volume of HepG2-M-derived tumors compared with HepG2-GAL1-derived tumors in mice treated with DOX at the end of the experiment (Fig. 2A–C). Final tumor volumes in DOX-treated versus untreated mice for HepG2-M-derived tumors were reduced 6.4-fold, whereas the response to treatment for HepG2-GAL1-derived tumors was significantly lower (2.4-folds) (Fig. 2C).

Notably, western blot analysis confirmed higher GAL1 levels in tumors derived from control mice inoculated with HepG2-GAL1 cells as compared to tumors derived from HepG2-M cells. Interestingly, a significant increase in GAL1 protein expression was found in DOX-treated tumor samples compared with non-treated tumor samples for each cell line (Fig. 2D), suggesting that tumor exposure to chemotherapeutic agents induces tumor GAL1 expression.

Altogether, these findings indicate that GAL1 overexpression protects HepG2-derived tumors from DOX treatment in vivo.

### GAL1-overexpressing HepG2 cells accumulate less intracellular DOX and exhibit increased P-gp protein levels

**Fig. 3:**
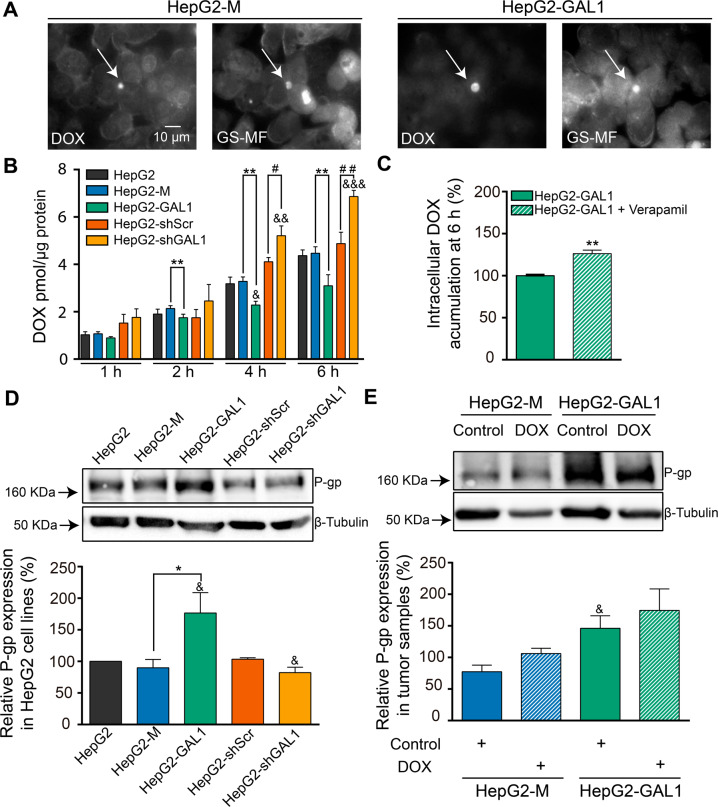
GAL1-overexpressing HepG2 cells accumulate less intracellular DOX and exhibit increased P-gp protein expression. **A** HepG2-M and HepG2-GAL1 cells were loaded with DOX (20 µM) and 5-chloromethylfluorescein diacetate (CMFDA) (2.5 µM). Metabolized glutathione methylfluorescein (GS-MF) was used to visualize canalicular structures. DOX and GS-MF fluorescence was registered in the same field. Ten coverslips/cell type were photographed, and representative images are shown (40X) (*n* = 3). Arrows indicate pseudocanaliculi that secreted both DOX and GS-MF. **B** HepG2 cells with different levels of GAL1 expression were exposed to DOX (20 µM) for the indicated times, and after treatment were scraped and lysed. The amount of DOX uptake by cells was determined with a fluorescence spectrophotometer using calibration curves. Total protein content in each sample was also measured. Results are expressed as the mean of DOX concentration (pmol/µg protein) ± SEM for each cell line and time point with respect to the accumulated by HepG2 cells after 1 h (*n* = 5). **p < 0.01 with respect to HepG2-M cells. ^*#*^*p* < 0.05, ^*##*^*p* < 0.01, with respect to HepG2-shScr. ^&^*p* < 0.05, ^&&^*p* < 0.01, ^&&&^*p* < 0.001, with respect to HepG2 cells. **C** Intracellular DOX accumulation in HepG2-GAL1 cells after 6 h treatment, with or without 20 µM verapamil (P-gp inhibitor). Results are expressed as the mean ± SEM of intracellular DOX accumulated by HepG2-GAL1 cells co-incubated with verapamil, with respect to DOX-treated HepG2-GAL1 cells in the absence of inhibitor (100%) (*n* = 4). **p < 0.01. **D** Western blot and densitometric analysis showing relative P-gp expression in GAL1-overexpressing, GAL1-silenced, and the corresponding control HepG2 cells. β-tubulin was used as loading control (*n* = 5). *p < 0.05 with respect to HepG2-M cells. ^&^*p* < 0.05 compared to HepG2 cells. **E** Relative P-gp protein expression in tumors derived from vehicle-treated (control) or DOX-treated mice inoculated with HepG2-M and HepG2-GAL1 cells (*n* = 5) (Fig. 2). ^&^*p* < 0.05 with respect to control HepG2-M-derived tumors.

The plasma membrane of hepatocytes is separated by tight junctions in canalicular (apical) and sinusoidal (basolateral) domains. In these cells, substances such as drugs are excreted into bile primarily by ABC transporters located at apical domains. HepG2 cells acquire a polarized phenotype characterized by the appearance of apical bile pseudocanaliculi between adjacent cells when cultured in vitro [23, 27]. To elucidate the mechanism underlying GAL1-driven chemoresistance in HCC cells, we investigated if HepG2 cells could excrete DOX to the bile pseudocanaliculi. We observed that fluorescent DOX co-localized with canalicular fluorescence of GS-MF in both HepG2-M and HepG2-GAL1 cells (Fig. 3A). These observations suggest that HepG2 cells excrete DOX to the bile pseudocanaliculi and that GAL1-overexpressing HepG2 cells maintain apical bile pseudocanaliculi secretory function of DOX.

Next, we investigated whether GAL1 influences intracellular DOX accumulation in HepG2 cells. After exposing cells to DOX we observed a significant decrease in intracellular drug concentration in HepG2-GAL1 cells compared with HepG2-M control cells. Further, knocking down GAL1 expression resulted in a significant increase in DOX concentration in HepG2-shGAL1 cells with respect to HepG2-shScr control cells (Fig. 3B).

As intracellular drug concentration is determined, in part, by ABC transporters and P-gp is involved in DOX efflux, we next co-incubated HepG2-GAL1 cells with DOX and verapamil, a pharmacological inhibitor of P-gp activity. Notably, inhibition of P-gp activity induced a significant increase in DOX accumulation in HepG2-GAL1 cells (Fig. 3C), suggesting the involvement of this transporter in reduction of drug concentration induced by GAL1 overexpression.

Next, we studied the effect of GAL1 on P-gp expression. We observed a significant increase in P-gp protein levels in HepG2-GAL1 cells compared with HepG2-M cells. On the contrary, silencing GAL1 expression did not alter the protein content of this transporter with respect to HepG2-shScr cells; however, P-gp levels in HepG2-shGAL1 cells were significantly reduced as compared with non-transfected HepG2 cells (Fig. 3D). Interestingly, in tumors grown in vivo following the chemotherapeutic regimen (Fig. 2), we found a tendency toward an increase in P-gp protein expression in tumors from DOX-treated mice compared with those from non-treated animals for each cell line (Fig. 3E). Although these differences were not statistically significant, our results suggest that chronic treatment with DOX could induce P-gp expression, leading to acquired MDR in HepG2-derived tumors. Furthermore, we observed a significant increase in P-gp levels in HepG2-GAL1-derived tumors respect to those generated by HepG2-M in control mice (Fig. 3E), indicating that overexpression of GAL1 in HepG2 cells influences expression of this transporter in vivo.

**Fig. 4:**
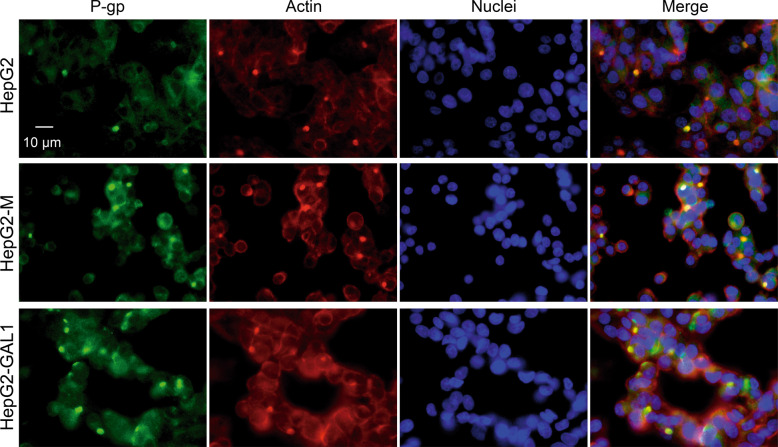
GAL1 overexpression does not alter the canalicular localization of P-gp in polarized HepG2 cells. P-gp is visualized in HepG2, HepG2-M, and HepG2-GAL1 cells by immunostaining and epifluorescence microscopy (green fluorescence) and apical bile pseudocanaliculi structures are identified by dense F-actin stained with TRITC-phalloidin (red fluorescence). Nuclei were stained with DAPI (40X). Photographs are representative of three independent experiments.

P-gp is localized at the canalicular or apical domain in polarized HepG2 cells. To evaluate if GAL1 overexpression could also alter P-gp localization, we performed immunofluorescence analysis of this transporter. Apical bile pseudocanaliculi structures were identified by dense F-actin stained with phalloidin in control and HepG2-GAL1 cells. We found that structures immunostained for P-gp were also stained with TRITC-phalloidin (Fig. 4), suggesting that P-gp localizes at the apical membrane surface in HepG2-GAL1 cells, similarly as in HepG2 and HepG2-M cells. Therefore, GAL1 overexpression does not alter sorting and transport of P-gp toward the apical membrane.

Collectively, these results demonstrate that heightened expression of GAL1 in HepG2 cells reduces intracellular accumulation of DOX likely by increasing P-gp protein expression rather than altering its membrane localization.

### GAL1-driven increase of P-gp expression may involve activation of the PI3K signaling pathway

**Fig. 5:**
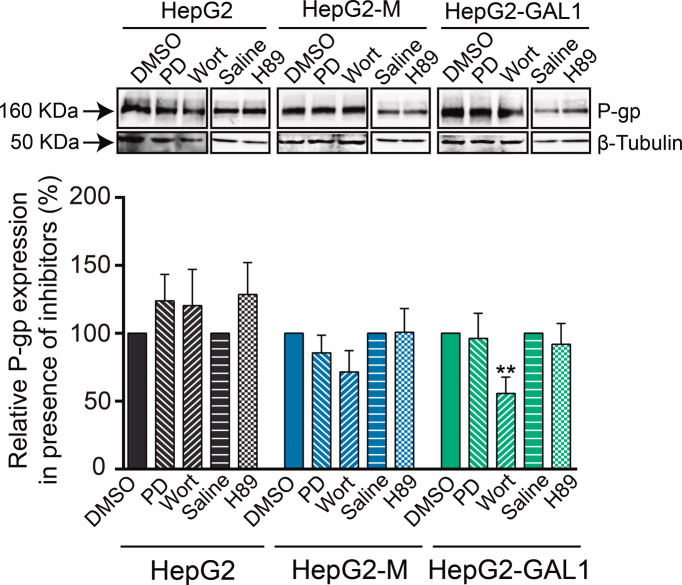
PI3K signaling pathway is involved in GAL1-driven increase of P-gp expression. P-gp expression analyzed by western blot (upper panel) and densitometric analysis (lower panel) in HepG2-GAL1 and control cells in the presence of signaling pathway inhibitors. Cells were pre-incubated for 24 h with PD98059 (PD, inhibitor of MEK), wortmannin (Wort, inhibitor of PI3K), and H89 (inhibitor of PKA). DMSO and saline solution were used as vehicles for PD98059 and wortmannin, and H89, respectively. β-tubulin was used as loading control. Results are expressed as the mean of relative P-gp expression in the presence of the indicated inhibitor with respect to the corresponding vehicle-treated cell lines (100%) ± SEM (*n* = 4). **p < 0.01 with respect to DMSO-treated HepG2-GAL1 cells.

Several signaling pathways are involved in the activation of the *MDR1* gene and the consequent increase in P-gp protein levels inducing a MDR phenotype [39]. Thus, we analyzed P-gp expression in HepG2-GAL1 and control cells in the presence of pharmacological inhibitors capable of interrupting these pathways. Treatment with the PI3K inhibitor wortmannin (1 µM) significantly decreased P-gp expression in HepG2-GAL1 cells as compared to vehicle-treated HepG2-GAL1 cells (Fig. 5). On the contrary, wortmannin did not produce any significant effect on P-gp expression in HepG2-M and HepG2 cells with respect to the corresponding vehicle-treated cells. Besides, we observed no effect on P-gp expression after treatment with the MEK inhibitor PD98059 or the PKA inhibitor H89 on the three cell lines analyzed (Fig. 5). This result suggests that PI3K signaling pathway mediates GAL1-driven increase of P-gp protein expression.

### P-gp is involved in GAL1-driven resistance to DOX in HepG2 cells

**Fig. 6:**
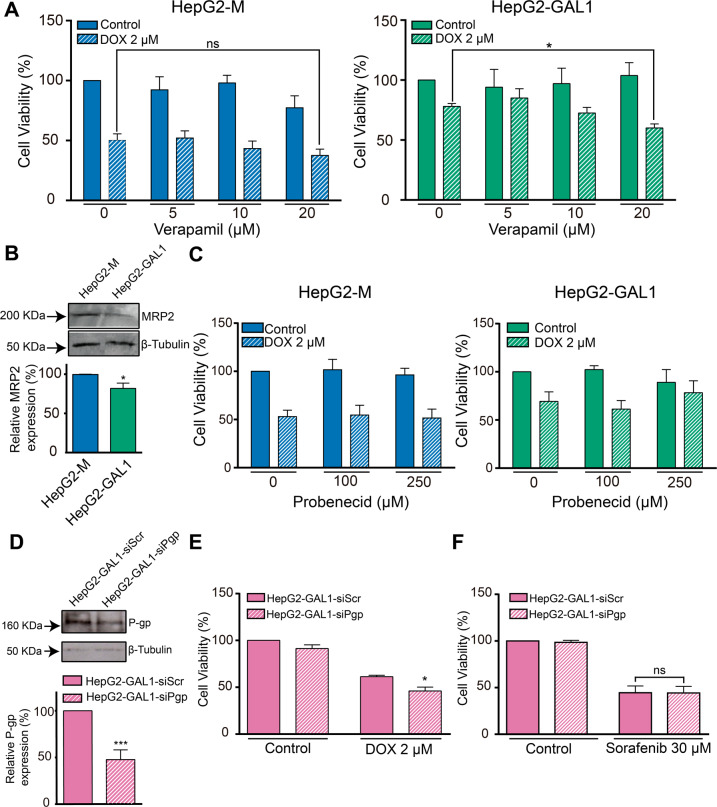
P-gp is involved in GAL1-mediated resistance to DOX in HepG2 cells. Cell viability (MTT assay) of GAL1-overexpressing and HepG2-M cells co-incubated with 2 µM DOX and **A** verapamil (P-gp inhibitor) or **C** probenecid (MRP2 inhibitor) at the indicated concentrations for 24 h. Results are expressed as the mean of cell viability percentage with respect to the corresponding untreated cell line (100%) ± SEM (*n* = 4). ns: no significant difference. *p < 0.05 compared with DOX-treatment HepG2-GAL1 cells without verapamil. **B** Western blot and densitometric analysis showing relative MRP2 expression in GAL1-overexpressing and control HepG2 cells. β-tubulin was used as loading control (*n* = 4). *p < 0.05 with respect to HepG2-M cells. **D** Western blot and densitometric analysis showing relative P-gp expression in HepG2-GAL1 cells after transfection with siRNA to specifically knockdown this transporter (HepG2-GAL1-siPgp cells) or with scrambled siRNA as control (HepG2-GAL1-siScr cells). β-tubulin was used as loading control (*n* = 4). ***p < 0.001 with respect to control cells. **E**, **F** Cell viability (MTT assay) in HepG2-GAL1-siScr and HepG2-GAL1-siPgp cells incubated in the absence (control) or the presence of DOX (2 µM) (**E**) or sorafenib (30 µM) (**F**) for 24 h. Results are expressed as the mean of cell viability percentage with respect to untreated siRNA scrambled transfected cells (100%) ± SEM (*n* = 5). *p < 0.05 with respect to DOX-treated HepG2-GAL1-siScr cells. ns: no significant difference.

To further confirm the role of P-gp in GAL1-mediated resistance to DOX, we developed ‘loss-of-function’ experiments by using the pharmacological inhibitor verapamil or by silencing the expression of the transporter. When we co-incubated HepG2-M cells with DOX and verapamil no significant changes in cell viability were observed with respect to cells incubated with DOX in the absence of the P-gp inhibitor (Fig. 6A). Remarkably, co-incubation of HepG2-GAL1 cells with DOX and verapamil significantly reduced cell viability compared with cells incubated with DOX alone (Fig. 6A).

MRP2, another member of the ABC transporter family, is upregulated in HCC patients [40] and determines the efficacy of cisplatin treatment [41]. To rule out the involvement of MRP2 in GAL1 effects on drug resistance, we analyzed its expression in GAL1-overexpressing cells. Remarkably, MRP2 protein levels were significantly decreased in HepG2-GAL1 cells with respect to HepG2-M cells (Fig. 6B). Moreover, although DOX has not been reported to be a substrate of MRP2 transporter, we used probenecid, a MRP2 inhibitor, as a control. As expected, we observed that probenecid did not affect DOX-treated HepG2-M or HepG2-GAL1 cell viability (Fig. 6C). Therefore, GAL1 overexpression protects HepG2 cells from cell death induced by DOX in a P-gp-, but not MRP2-dependent manner.

In addition, we silenced P-gp expression in GAL1-overexpressing cells. HepG2-GAL1-siPgp cells exhibited a 53% decrease in P-gp protein expression with respect to HepG2-GAL1-siScr control cells (Fig. 6D). Importantly, percentage of cell viability in HepG2-GAL1-siPgp cells was significantly reduced upon treatment with DOX with respect to HepG2-GAL1-siScr cells (Fig. 6E). On the contrary, no significant changes in cell viability were observed in HepG2-GAL1-siPgp cells with respect to control cells after incubation with sorafenib (Fig. 6F). Thus, P-gp silencing sensitizes HepG2-GAL1 cells to DOX-, but not to sorafenib-induced cell death.

**Fig. 7:**
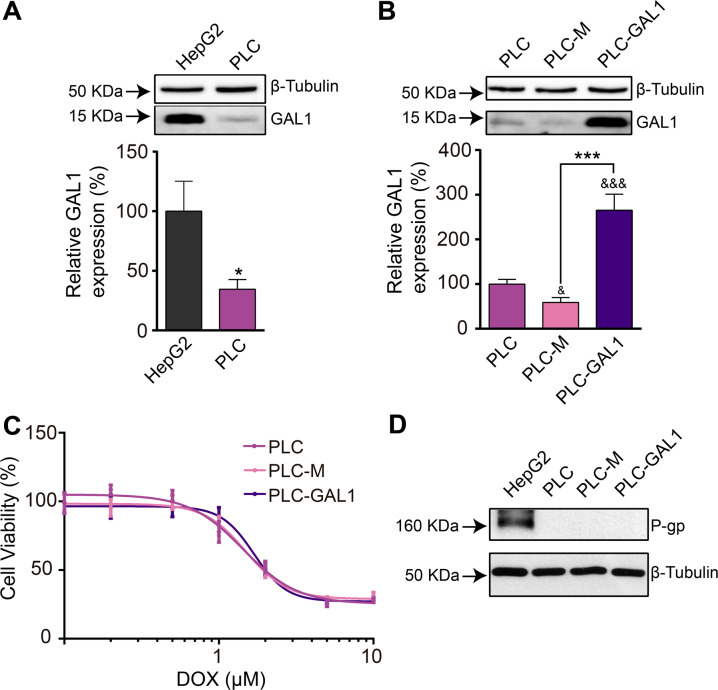
GAL-1 overexpression in PLC/PRF/5 HCC cells is not sufficient to protect cells from DOX-induced cell death. **A** Western blot and densitometric analysis showing relative GAL1 expression in PLC/PRF/5 (PLC) and HepG2 cell lysates. β-tubulin was used as loading control (*n* = 9). *p < 0.05 with respect to HepG2 cells. **B** Western blot and densitometric analysis showing relative GAL1 expression in non-transfected PLC/PRF/5 (PLC) and transfected with pcDNA3.1-*LGALS1* (PLC-GAL1) or expression vector without insert (PLC-M) cell lysates. β-tubulin was used as loading control (*n* = 7). ***p < 0.001 with respect to PLC-M cells. ^&&&^*p* < 0.001 with respect to PLC cells. **C** Cell viability (MTT assay) in GAL1-overexpressing and control cells incubated with increasing concentrations of DOX (0–10 µM) for 48 h. Results are expressed as the mean of cell viability percentage with respect to the corresponding untreated cell line (100%) ± SEM (*n* = 10). The experimental data were fitted to dose–response curves by non-linear regression as described in “Materials and methods”. **D** Western blot showing P-gp expression in HepG2, PLC/PRF/5 (PLC), control PLC-M, and GAL1-overexpressing (PLC-GAL1) cell lysates. β-tubulin was used as loading control (*n* = 5).

Next, we used another human HCC cell line to further study GAL1-driven resistance to DOX. GAL1 protein expression in PLC/PRF/5 cells demonstrated to be 65% lower than in HepG2 cells (Fig. 7A). Thus, we stably transfected PLC/PRF/5 cells with either GAL1 cDNA constructs (PLC/PRF/5-GAL1 cells) to overexpress this lectin or with expression vector alone as control (PLC/PRF/5-M cells). Although PLC/PRF/5-GAL1 cells showed approximately 2.6- and 4.5-fold higher expression of GAL1, with respect to non-transfected PLC/PRF/5 cells and PLC/PRF/5-M cells, respectively, (Fig. 7B), they were as sensitive as control cells to the cytotoxic effects of DOX (Fig. 7C). After 48 h treatment with DOX, GAL1-overexpressing cells displayed similar viability curves (Fig. 7C) as control cells. Similar results were obtained after sorafenib treatment (data not shown). Interestingly, when we analyzed P-gp protein levels we found that this drug transporter was undetectable in PLC/PRF/5 and PLC/PRF/5-M cells, and it remained undetectable even though GAL1 was overexpressed in PLC/PRF/5-GAL1 cells (Fig. 7D). Altogether, these results indicate that P-gp mediates GAL1-driven resistance to DOX in HepG2 cells. Moreover, in PLC/PRF/5 cells which showed undetectable P-gp protein expression, GAL1 does not control resistance to DOX or sorafenib, reinforcing the idea that GAL1 effects are mediated by P-gp.

## Discussion

Mounting evidence indicates that GAL1 is a key determinant of chemoresistance developed by different tumor types [42–50]. Strikingly, although increased levels of GAL1 are a hallmark of HCC progression, aggressiveness, and metastasis [20, 21], limited information is available on the role of this lectin in HCC chemoresistance. Besides, the precise mechanisms underlying this effect are not completely elucidated. Yeh et al. described that GAL1 is upregulated in HuH-7 HCC cells that acquired resistance to sorafenib (HuH-7R) and in HuH-7R-derived xenograft tumors with respect to parental cells. In addition, increased GAL1 expression in HCC patients’ serum or tumor tissue was associated with low efficacy to sorafenib treatment and poor survival outcome [31, 32]. In this study, we demonstrated that GAL1 overexpression protects HepG2 cells from DOX- and sorafenib-induced cell death in vitro. Moreover, we showed that increased levels of this lectin in HepG2 cells generate DOX-resistant tumors, demonstrating for the first time the involvement of GAL1 in HCC chemoresistance in vivo. Our findings also revealed that DOX treatment induces tumor GAL1 expression. Thus, taken together these results indicate that GAL1 is involved in both intrinsic and acquired resistance to DOX and sorafenib in HCC cells.

One of the molecular mechanisms underlying HCC chemoresistance involves modulation of intracellular drug concentration determined, in part, by ABC transporters [10, 11]. As P-gp is involved in DOX efflux, we investigated its contribution in GAL1-mediated chemoresistance in HepG2 cells. We observed that cells that overexpress this galectin accumulate less intracellular DOX and exhibit increased P-gp protein levels without altering its apical membrane localization. Furthermore, we showed evidence that P-gp is involved in GAL1-mediated resistance to DOX in HepG2 cells. These findings reveal a possible mechanism through which GAL1 protects these cells from DOX-induced death.

MRP2 has been demonstrated to be upregulated in HCC patients [40] and its overexpression determines the efficacy of cisplatin treatment [41]. Of note, we observed that MRP2 expression decreased in HepG2-GAL1 cells. Moreover, our results indicate that GAL1 overexpression protects HepG2 cells from DOX-induced cell death via an MRP2-independent manner. These findings added to the fact that DOX is not likely a substrate of MRP2 transporter, ruled out the involvement of MRP2 in GAL1-driven resistance to DOX, at least in HepG2 cells.

Our results also showed that silencing P-gp expression did not sensitize HepG2-GAL1 cells to sorafenib-induced cell death, suggesting that this transporter is not involved in GAL1-driven resistance to sorafenib in these cells. Sorafenib is a weak substrate of P-gp and interestingly, some studies highlight its role in decreasing ABC transporter expression. After 48 h incubation with this drug, DOX-resistant HepG2 cells decreased P-gp protein expression and became more sensitive to cell death [51]. Conversely, other studies showed that P-gp, MRP2, multidrug resistance-associated protein 1 (MRP1), and ABC subfamily G member 2 (ABCG2) are key resistance factors that control the pharmacokinetics and pharmacodynamics of sorafenib in HCC [52]. Thus, further work is required to elucidate the involvement of ABC transporters in GAL1-induced resistance to sorafenib in HCC cells.

Within the extracellular milieu, GAL1 can regulate cell adhesion and migration, tumor growth, angiogenesis, tumor-immune escape, and metastasis through binding to specific glycans on the cell surface or extracellular matrix [15–17]. Moreover, this lectin can also act intracellularly by interacting with the RAS binding domain of RAF effectors and increasing H-RAS nanoclustering, driving tumor transformation [53]. In this regard, we previously described that HepG2-derived GAL1 is responsible for inducing E-cadherin downregulation and thus, EMT through extracellular ligand-independent mechanisms [27]. Moreover, concerning ABC transporters, we previously reported that extracellular recombinant GAL1 induced no effects on P-gp or MRP2 protein expression [23]. Remarkably, in this work, we found that HepG2-GAL1 cells display altered levels of these transporters, as compared to control cells. Hence, high levels of intracellular GAL1 are responsible for inducing P-gp upregulation and MRP2 downregulation in HepG2 cells. Nam et al. found that binding of extracellular GAL-1 to integrin β_1_ enhances drug resistance by promoting survivin expression in breast cancer cells [47]. Thus, we cannot rule out the possibility that GAL1 might trigger additional extracellular mechanisms of chemoresistance in HCC cells.

We observed that the PI3K signaling pathway is involved in GAL1-induced increase of P-gp expression in HepG2 cells. Several signaling pathways control the activation of the *MDR1* gene and the consequent increase in P-gp protein levels, inducing a MDR phenotype. Among the most relevant are MAPK, PI3K, PKA, and protein kinase C (PKC) signaling pathways [38]. Notably, PI3K and RAF-1/MAPK/ERK signaling pathways are central routes downstream of H-RAS, an oncogenic protein activated by GAL1 [54, 55]. In this sense, we previously showed critical effects of GAL1 on HCC cell adhesion and polarization as well as E-cadherin expression that were also mediated by PI3K signaling [23, 27].

To expand our findings, we then used PLC/PRF/5 cells, another human well-differentiated HCC cell line. We observed that GAL1 protein expression in these cells was significantly lower than in HepG2 cells. Interestingly, our results showed that PLC/PRF/5 cells do not express P-gp and that GAL1 overexpression in PLC/PRF/5 cells does not protect them from DOX- or sorafenib-induced cell death and does not increase P-gp protein expression to detectable levels. We hypothesize that GAL1-mediated DOX resistance requires a functional *MDR1* gene and this effect does not seem to occur in PLC/PRF/5 cells. In fact, some reports showed undetectable P-gp levels in western blot experiments in PLC/PRF/5 cells, while others showed low protein levels; however, P-gp protein expression increased after cells acquired drug resistance [56–58]. Epigenetic processes, such as DNA methylation and post-transcriptional histone modifications, are involved in P-gp-mediated drug resistance [59]. Thus, the different effects observed in HepG2 and PLC/PRF/5 cells regarding GAL1, P-gp expression, and drug resistance could be related to the different functional status of *MDR1* gene in both cell lines. Moreover, we propose that the transcription of *MDR1* gene in PLC/PRF/5 cells may not be active, probably because of epigenetic modifications at the *MDR1* gene promoter. Hence, GAL1 upregulation in PLC/PRF/5 cells will not increase the expression levels of P-gp mRNA, and resistance to DOX will not take place.

Several microRNAs were found to participate in *MDR1* gene regulation [59]. Importantly, many of them are downregulated in human HCC tissues and cell lines. Consequently, the loss of miRNA-dependent post-transcriptional control leads to elevated P-gp protein levels and the acquisition of resistance to DOX-induced HCC cell death [60]. Of note, miR-22 level is also significantly lower in HCC tumor tissues than in normal samples [61, 62] and interestingly, miR-22 silencing induced GAL1 upregulation and enhanced HCC cell growth, migration, and invasion [63]. Therefore, the different effects observed in HepG2 and PLC/PRF/5 cells regarding GAL1 and P-gp protein expression, and resistance to DOX and sorafenib could also be related to the altered expression of different miRNAs in each cell line.

Our previous results revealed that GAL1 overexpression promotes EMT in HepG2 cells inducing the expression of the transcription factor SNAIL and activating the Wnt pathway [27]. Here, we demonstrated that increased expression of this lectin also confers drug resistance in these cells. In line with our findings, several signaling pathways known to induce EMT, such as TGF-β, Wnt, Hedgehog, and Notch may also induce resistance to anticancer drugs [28]. Moreover, transcription factors known to trigger EMT such as TWIST, SNAIL, and FOXC2 increase the expression of ABC transporters in breast cancer [64, 65]. In addition, activated TWIST mediates P-gp expression in bladder cancer [66]. Remarkably, SNAIL1 directly upregulates *MDR1* gene transcription inducing drug resistance, and post-transcriptionally inhibited P53 protein expression through hsa-miRNA-22-3p, inhibiting apoptosis in multiple myeloma cells [67]. Thus we could speculate that the differences observed between both cell lines in response to GAL1 might also be related to the P53 status. HepG2 cells express wild-type P53, whereas PLC/PRF/5 cells express mutant P53 (according to ATCC and ref. [68]). Thus, wild-type P53 expression may be also important in GAL1-induced drug resistance in HCC cells.

In conclusion, our results show that GAL1 protects HCC HepG2 cells from DOX- and sorafenib-induced cell death. Mechanistically, GAL1-overexpressing HepG2 cells accumulate less intracellular DOX likely due to increased P-gp expression. Moreover, this transporter plays an important role in GAL1-induced resistance to DOX. Collectively, these findings provide new insights into the chemoresistance mechanisms triggered by GAL1, emphasizing the role of this lectin as a potential therapeutic target in HCC.

## Supplementary information

Checklist

Author contribution form

Pre-authorship form

Authorship agreement.

## Data Availability

The data generated or analyzed are included in this article.
