# Supplementary material for: Galectin-1 confers resistance to doxorubicin in hepatocellular carcinoma cells through modulation of P-glycoprotein expression
Source: Cell Death Dis. 2022 Jan 24;13(1):79. doi: 10.1038/s41419-022-04520-6 (PMC8786848; doi:10.1038/s41419-022-04520-6)
Supplement: Supplementary file 2 — Author contribution form [file 41419_2022_4520_MOESM2_ESM.pdf]

DECLARATION OF CONTRIBUTIONS TO ARTICLE

ADMC

Manuscript Number: CDDIS-21-0674R

Journal Name: *Cell Death & Disease* (the 'Journal')

Proposed Title of the Contribution:

Galectin-1 confers resistance to doxorubicin in hepatocellular carcinoma cells through modulation of P-glycoprotein expression (the 'Contribution)

Author(s):

Pablo Carabias, María V. Espelt, María L. Bacigalupo, Paola Rojas, Luciana Sarrias, Ayelén Rubin, Nicolás A. Saffioti, María T. Elola, Juan P. Rossi, Carlota Wolfenstein-Todel, Gabriel A. Rabinovich, María F. Troncoso (the 'Authors')

For all *CDDis* articles, each person named as an author in the published version must be able to show he or she has contributed substantially to the article.

Authorship credit should be based on 1) substantial contributions to conception and design, acquisition of data, or analysis and interpretation of data; 2) drafting the article or revising it critically for important intellectual content; and 3) final approval of the version to be published. Authors should meet conditions 1, 2 and 3.

Any person who cannot be shown to have made a substantial contribution to the article cannot be listed as an author in the final version. The name of any person who is deemed to have made a minor contribution can, however, appear in the Acknowledgments section of the article.

Please complete the table below to indicate the contributions of all named authors to the manuscript.

Author Full Name:	Specification of Contribution to the Manuscript:
Pablo Carabias	Design, acquisition, analysis and interpretation of data; drafting and revising the manuscript; approval of the final version
María V. Espelt	Design, acquisition, analysis and interpretation of data; drafting and revising the manuscript; approval of the final version
María L. Bacigalupo	Acquisition, analysis and interpretation of data; revising the manuscript; approval of the final version
Paola Rojas	Design, acquisition, analysis and interpretation of data; drafting and revising the manuscript; approval of the final version
Luciana Sarrias	Acquisition, analysis and interpretation of data; revising the manuscript; approval of the final version
Ayelén Rubin	Acquisition, analysis and interpretation of data; revising the manuscript; approval of the final version
Nicolás A. Saffioti	Acquisition, analysis and interpretation of data; revising the manuscript; approval of the final version
María T. Elola	Interpretation of data, revising the manuscript critically for important intellectual content, approval of the final version
Juan P. Rossi	Interpretation of data, revising the manuscript, approval of the final version
Carlota Wolfenstein-Todel	Interpretation of data, revising the manuscript, approval of the final version
Gabriel A. Rabinovich	Interpretation of data, revising the manuscript critically for important intellectual content, approval of the final version
María F. Troncoso	Conception and design, analysis and interpretation of data; drafting and revising the manuscript; approval of the final version

Please complete the table below to indicate the contributions of all named authors to the figures.

Figure 1:

Pablo Carabias, María L. Bacigalupo, Carlota Wolfenstein- Todel, María F. Troncoso

Figure 2:

Pablo Carabias, Paola Rojas, Ayelén Rubin, Gabriel A. Rabinovich, María F. Troncoso

Figure 3:

Pablo Carabias, María V. Espelt, Nicolás A. Saffioti, Juan P. Rossi, Paola Rojas, María F. Troncoso

Figure 4:

Pablo Carabias, María V. Espelt, María L. Bacigalupo, María F. Troncoso

Figure 5:

Pablo Carabias, María L. Bacigalupo, María F. Troncoso

Figure 6:

Pablo Carabias, María V. Espelt, María L. Bacigalupo, Luciana Sarrias, María F. Troncoso

Figure 7:
María V. Espelt, Luciana Sarrias, María T. Elola, María F. Troncoso

Signed for and on behalf of the Author(s):	Print Name:	Date:
	María Fernanda Troncoso	11-17-2021
