# Supplementary material for: Galectin-1 confers resistance to doxorubicin in hepatocellular carcinoma cells through modulation of P-glycoprotein expression
Source: Cell Death Dis. 2022 Jan 24;13(1):79. doi: 10.1038/s41419-022-04520-6 (PMC8786848; doi:10.1038/s41419-022-04520-6)
Supplement: Supplementary file 3 — Pre-authorship form [file 41419_2022_4520_MOESM3_ESM.pdf]

SPRINGER NATURE

Change of authorship request form - Journals (pre-acceptance)

Important information. Please read.

- This form should be used by authors to request any change in authorship (adding/deleting authors) including changes in corresponding authors. This form should not be used for name changes. Please fully complete all sections. Use black ink and block capitals and provide each author's full name with the given name first followed by the family name.

- By signing this declaration, all authors guarantee that the order of the authors are in accordance with their scientific contribution, if applicable as different conventions apply per discipline, and that only authors have been added who made a meaningful contribution to the work.

- Please note, in author collaborations where there is formal agreement for representing the collaboration, it is sufficient for the representative or legal guarantor (usually the corresponding author) to complete and sign the Authorship Change Form on behalf of all authors, **next to the added/removed author(s)**. **(Complete Section 3, followed by Section 6.)**
  In author collaborations where there is no formal agreement for representing the collaboration and **there are more than 10 authors**, one may sign for all, provided the signer appends correspondence that attests that each of the authors have agreed to the change **and the added/removed authors sign the form**. **(Complete Section 3, followed by Section 6.)**

- Please note, we cannot investigate or mediate any authorship disputes. If you are unable to obtain agreement from all authors (including those who you wish to be removed) you must refer the matter to your institution(s) for investigation. Please inform us if you need to do this.

- If you are not able to return a fully completed form within **30 days** of the date that it was sent to the author requesting the change, we may have to withdraw your manuscript. We cannot publish manuscripts where authorship has not been agreed by all authors (including those who have been removed).

- Incomplete forms will be rejected.

- Please return/upload this form, fully completed, to the Journals Editorial Office. The Journal and/or Publisher will consider the information you have provided to decide whether to approve the proposed change in authorship. We may decide to contact your institution for more information or undertake a further investigation, if appropriate, before making a final decision.

SPRINGER NATURE Springer Nature is one of the world's leading global research, educational and professional publishers, created in May 2015 through the combination of Nature Publishing Group, Palgrave Macmillan, Macmillan Education and Springer Science+Business Media.

SPRINGER NATURE

Change of authorship request form - Journals (pre-acceptance)

Section 1: Please provide the current title of manuscript

Manuscript ID no.: CDDIS-21-0674R

Title: Galectin-1 confers resistance to doxorubicin in hepatocellular carcinoma cells through modulation of P-glycoprotein expression

Section 2: Please provide the previous authorship, in the order shown on the manuscript before the changes were introduced. Please indicate the corresponding author by adding (CA) behind the name.

	First name(s)	Family name	ORCID or SCOPUS id, if available
1st author	Pablo	Carabias	
2nd author	María Lorena	Bacigalupo	
3rd author	Paola	Rojas	
4th author	Ayelén	Rubin	
5th author	Nicolás Andrés	Saffioti	
6th author	María Teresa	Elola	
7th author	Juan Pablo	Rossi	
8th author	Carlota	Wolfenstein-Todel	
9th author	María Victoria	Espelt	
10th author	Gabriel Adrián	Rabinovich	

Please use an additional sheet if there are more than 10 authors.

SPRINGER NATURE Springer Nature is one of the world's leading global research, educational and professional publishers, created in May 2015 through the combination of Nature Publishing Group, Palgrave Macmillan, Macmillan Education and Springer Science+Business Media.

SPRINGER NATURE Change of authorship request form - Journals (pre-acceptance)

Section 1: Please provide the current title of manuscript

Manuscript ID no.: CDDIS-21-0674R

Title: Galectin-1 confers resistance to doxorubicin in hepatocellular carcinoma cells through modulation of P-glycoprotein expression

Section 2: Please provide the previous authorship, in the order shown on the manuscript before the changes were introduced. Please indicate the corresponding author by adding (CA) behind the name.

	First name(s)	Family name	ORCID or SCOPUS id, if available
11 ~~1~~st author	María Fernanda	Troncoso	0000-0002-4919-9976
2nd author			
3rd author			
4th author			
5th author			
6th author			
7th author			
8th author			
9th author			
10th author			

Please use an additional sheet if there are more than 10 authors.

SPRINGER NATURE Springer Nature is one of the world's leading global research, educational and professional publishers, created in May 2015 through the combination of Nature Publishing Group, Palgrave Macmillan, Macmillan Education and Springer Science+Business Media.

SPRINGER NATURE

Change of authorship request form - Journals (pre-acceptance)

Section 3: Please provide a justification for change. Please use this section to explain your reasons for changing the authorship of your manuscript, e.g. what necessitated the change in authorship? Please refer to the (journal) policy pages for more information about authorship. Please explain why omitted authors were not originally included and/or why authors were removed on the submitted manuscript.

Additional experiments requested by reviewers were performed by Luciana Sarrias and María Victoria Espelt.
Thus, Luciana Sarrias was included as a coauthor, and María Victoria Espelt was moved to the second place in the list of authors.

Section 4: Proposed new authorship. Please provide your new authorship list in the order you would like it to appear on the manuscript. Please indicate the corresponding author by adding (CA) behind the name. <u>If the Corresponding Author has changed, please indicate the reason under section 3</u>.

	First name(s)	Family name (this name will appear in full on the final publication and will be searchable in various abstract and indexing databases)	Affiliated institute	E-mail address
1st author	Pablo	Carabias	UBA. CONICET.	carabiaspablo@gmail.com
2nd author	María Victoria	Espelt	UBA. CONICET.	vicespelt@gmail.com
3rd author	María Lorena	Bacigalupo	UBA. CONICET.	lorebaci@gmail.com
4th author	Paola	Rojas	CONICET	parojas2010@gmail.com
5th author	Luciana	Sarrias	UBA. CONICET.	lusarrias13@gmail.com
6th author	Ayelén	Rubin	CONICET	aye.rubin@gmail.com
7th author	Nicolás Andrés	Saffioti	UBA. CONICET.	nsaffioti@gmail.com
8th author	María Teresa	Elola	UBA. CONICET.	mt_elola@yahoo.com
9th author	Juan Pablo	Rossi	UBA. CONICET.	jprossi@qb.ffyb.uba.ar
10th author	Carlota	Wolfenstein-Todel	UBA. CONICET.	cwtodel2002@yahoo.com.ar

Please use an additional sheet if there are more than 10 authors.

SPRINGER NATURE Springer Nature is one of the world's leading global research, educational and professional publishers, created in May 2015 through the combination of Nature Publishing Group, Palgrave Macmillan, Macmillan Education and Springer Science+Business Media.

SPRINGER NATURE

Change of authorship request form - Journals (pre-acceptance)

Section 3: Please provide a justification for change. Please use this section to explain your reasons for changing the authorship of your manuscript, e.g. what necessitated the change in authorship? Please refer to the (journal) policy pages for more information about authorship. Please explain why omitted authors were not originally included and/or why authors were removed on the submitted manuscript.

Section 4: Proposed new authorship. Please provide your new authorship list in the order you would like it to appear on the manuscript. Please indicate the corresponding author by adding (CA) behind the name. <u>If the Corresponding Author has changed, please indicate the reason under section 3</u>.

	First name(s)	Family name (this name will appear in full on the final publication and will be searchable in various abstract and indexing databases)	Affiliated institute	E-mail address
11 ~~1~~st author	Gabriel Adrián	Rabinovich	CONICET. UBA.	gabyrabi@gmail.com
12 ~~2~~nd author	María Fernanda	Troncoso	UBA. CONICET	fernanda@qb.ffyb.uba.ar / ma.f.troncoso@gmail.com
3rd author				
4th author				
5th author				
6th author				
7th author				
8th author				
9th author				
10th author				

Please use an additional sheet if there are more than 10 authors.

SPRINGER NATURE Springer Nature is one of the world's leading global research, educational and professional publishers, created in May 2015 through the combination of Nature Publishing Group, Palgrave Macmillan, Macmillan Education and Springer Science+Business Media.

SPRINGER NATURE

Change of authorship request form - Journals (pre-acceptance)

Section 5: Author contribution, Acknowledgement and Disclosures. Please use this section to provide a new disclosure statement and, if appropriate, acknowledge any contributors who have been removed as authors and ensure you state what contribution any new authors made (if applicable per the journal or book (series) policy). Please ensure these are updated in your manuscript - after approval of the change(s) - as our production department will not transfer the information in this form to your manuscript.

New acknowledgements:

PC, MLB, AR and NAS received fellowships from Consejo Nacional de Investigaciones Científicas y Técnicas (CONICET).
MLB was a researcher at the Universidad de Buenos Aires.
LS obtained a fellowship from Ministerio de Ciencia, Tecnología e Innovación, Agencia Nacional de Promoción de la Investigación, el Desarrollo Tecnológico y la Innovación.
MVE, PR, MTE, JPR, CWT, GAR and MFT are researchers from CONICET.

New Disclosures (financial and non-financial interests, funding):

New Author Contributions statement (if applicable per the journal policy):

PC and MVE performed the experiments, analyzed data and wrote and revised the manuscript.
MLB, LS, AR and NAS performed experiments and analyzed data.
PR designed and performed experiments, and analyzed data.
MTE provided critical feedback and edited the manuscript.
JPR and CWT provided material support and reviewed the manuscript.
GAR provided material support, and edited and critically revised the manuscript for important intellectual content.
MFT conceived the idea, devised the study, oversaw the research program, analyzed data, and wrote and revised the manuscript.
All authors read and approved the final manuscript.

State 'Not applicable' if there are no new authors.

SPRINGER NATURE Springer Nature is one of the world's leading global research, educational and professional publishers, created in May 2015 through the combination of Nature Publishing Group, Palgrave Macmillan, Macmillan Education and Springer Science+Business Media.

SPRINGER NATURE

Change of authorship request form - Journals (pre-acceptance)

Section 6: Declaration of agreement. *All* authors, unchanged, new and removed *must* sign this declaration.
(NB: Please print the form, (docu)-sign and return/upload a scanned copy. Please note that signatures that have been inserted as an image file are acceptable as long as it is handwritten. Typed names in the signature box are unacceptable.) *** Please delete as appropriate. Delete all of the bold if you were on the original authorship list and are remaining as an author.**

	First name	Family name		Signature	Date
1st author	Pablo	Carabias	I agree to the proposed new authorship shown in section 4 /~~**and the addition/removal*of my name to the authorship list**~~ /~~and the proposed change in corresponding author~~		11-17-2021
2nd author	María Victoria	Espelt	I agree to the proposed new authorship shown in section 4 /~~**and the addition/removal*of my name to the authorship list**~~ /~~and the proposed change in corresponding author~~		11-17-2021
3rd author	María Lorena	Bacigalupo	I agree to the proposed new authorship shown in section 4 /~~**and the addition/removal*of my name to the authorship list**~~ /~~and the proposed change in corresponding author~~		11-17-2021
4th authors	Paola	Rojas	I agree to the proposed new authorship shown in section 4 /~~**and the addition/removal*of my name to the authorship list**~~ /~~and the proposed change in corresponding author~~		11-17-2021
5th author	Luciana	Sarrias	I agree to the proposed new authorship shown in section 4 /**and the addition/~~removal~~*of my name to the authorship list** /~~and the proposed change in corresponding author~~		11-17-2021
6th author	Ayelén	Rubin	I agree to the proposed new authorship shown in section 4 /~~**and the addition/removal*of my name to the authorship list**~~ /~~and the proposed change in corresponding author~~		11-17-2021
7th author	Nicolás Andrés	Saffioti	I agree to the proposed new authorship shown in section 4 /~~**and the addition/removal*of my name to the authorship list**~~ /~~and the proposed change in corresponding author~~		11-17-2021

SPRINGER NATURE Springer Nature is one of the world's leading global research, educational and professional publishers, created in May 2015 through the combination of Nature Publishing Group, Palgrave Macmillan, Macmillan Education and Springer Science+Business Media.

SPRINGER NATURE

Change of authorship request form - Journals (pre-acceptance)

	First name	Family name		Signature	Date
8th author	María Teresa	Elola	I agree to the proposed new authorship shown in section 4 ~~/**and the addition/removal*of my name to the authorship list** /and the proposed change in corresponding author~~		11-17-2021
9th author	Juan Pablo	Rossi	I agree to the proposed new authorship shown in section 4 ~~/**and the addition/removal*of my name to the authorship list** /and the proposed change in corresponding author~~		11-17-2021
10th author	Carlota	Wolfenstein-Todel	I agree to the proposed new authorship shown in section 4 ~~/**and the addition/removal*of my name to the authorship list** /and the proposed change in corresponding author~~		11-17-2021

Please use an additional sheet if there are more than 10 authors.

In case of author collaborations with formal agreement:

	Name of consortium/consortia	First name	Family name		Signature	Date
Representative/legal guarantor				I agree to the proposed new authorship shown in section 4 /**and the addition/removal*of my name to the authorship list** /and the proposed change in corresponding author		

Both added/removed authors should complete the information in the first table under Section 6.

---- End of form ----

SPRINGER NATURE Springer Nature is one of the world's leading global research, educational and professional publishers, created in May 2015 through the combination of Nature Publishing Group, Palgrave Macmillan, Macmillan Education and Springer Science+Business Media.

SPRINGER NATURE

Change of authorship request form - Journals (pre-acceptance)

	First name	Family name		Signature	Date
11 ~~8~~th author	Gabriel Adrián	Rabinovich	I agree to the proposed new authorship shown in section 4 /~~**and the addition/removal*of my name to the authorship list** /and the proposed change in corresponding author~~		11-17-2021
12 ~~9~~th author	María Fernanda	Troncoso	I agree to the proposed new authorship shown in section 4 /~~**and the addition/removal*of my name to the authorship list** /and the proposed change in corresponding author~~		11-17-2021
10th author			I agree to the proposed new authorship shown in section 4 /**and the addition/removal*of my name to the authorship list** /and the proposed change in corresponding author		

Please use an additional sheet if there are more than 10 authors.

In case of author collaborations with formal agreement:

	Name of consortium/consortia	First name	Family name		Signature	Date
Representative/legal guarantor				I agree to the proposed new authorship shown in section 4 /**and the addition/removal*of my name to the authorship list** /and the proposed change in corresponding author		

Both added/removed authors should complete the information in the first table under Section 6.

---- End of form ----

SPRINGER NATURE Springer Nature is one of the world's leading global research, educational and professional publishers, created in May 2015 through the combination of Nature Publishing Group, Palgrave Macmillan, Macmillan Education and Springer Science+Business Media.
