# Supplementary material for: Galectin-1 confers resistance to doxorubicin in hepatocellular carcinoma cells through modulation of P-glycoprotein expression
Source: Cell Death Dis. 2022 Jan 24;13(1):79. doi: 10.1038/s41419-022-04520-6 (PMC8786848; doi:10.1038/s41419-022-04520-6)
Supplement: Supplementary file 4 — Authorship agreement. [file 41419_2022_4520_MOESM4_ESM.pdf]

CDDIS-21-0674RR Initial Quality | Gmail - CDDIS-21-0674RR Initial

mail.google.com/mail/u/0/?ik=94e8bbcc0f&view=pt&search=all&permthid=thread-f%3A1721296325383708665&dsqt=1&simpl=msg-f%3A1721296325383708665&si...

Aplicaciones | Primeros pasos | Últimas noticias | Importado desde Fi... | Medical Journals | Harvard University... | SJR : Scientific Jour... | LGALS1 galectin 1 [... | Lista de lectura

María Fernanda Troncoso <ma.f.troncoso@gmail.com> 7 de enero de 2022, 12:00

Para: Pablo Carabias <carabiaspablo@gmail.com>, Maria Victoria Espelt <vicespelt@gmail.com>, María Lorena Bacigalupo <lorebaci@gmail.com>, Paola Rojas <parojas2010@gmail.com>, Luciana Sarrias <lusarrias13@gmail.com>, aye.rubin@gmail.com, Nicolas Andres Saffioti <nsaffioti@gmail.com>, maria teresa elola <mt_elola@yahoo.com>, Jean Paul Rossi <jprossi@qb.ffyb.uba.ar>, Vonckers <vonckers@gmail.com>, Carlota Wolfenstein de Todel <cwtodel2002@yahoo.com.ar>, Gaby Rabinovich <gabyrabi@gmail.com>

Dear co-authors,

As requested by Cell Death and Disease, I would like to ask you to reply my email confirming that you agree with the new authorships of our manuscript:

Manuscript Number: CDDIS-21-0674RR

Title: Galectin-1 confers resistance to doxorubicin in hepatocellular carcinoma cells through modulation of P-glycoprotein expression

Authors: Pablo Carabias*, María Espelt*, María Bacigalupo, Paola Rojas, Luciana Sarrias, Ayelén Rubin, Nicolás Saffioti, María Elola, Juan Rossi, Carlota Wolfenstein-Todel, Gabriel Rabinovich, and María Troncoso

Pablo Carabias and María V Espelt contributed equally to this work
Luciana Sarrias was incorporated as co-author

Thanks!

Best wishes
Fernanda

---------- Forwarded message ---------
De: <cddisease@springernature.com>
Date: vie, 7 ene 2022 a las 8:48
Subject: CDDIS-21-0674RR Initial Quality Check
To: <ma.f.troncoso@gmail.com>

Dear Dr Troncoso,

In checking in your manuscript submitted to Cell Death & Disease it has come to our attention that the following must be addressed before we can proceed.

06:20 p.m.
07/01/2022

CDDIS-21-0674RR Initial Quality (×   Gmail - CDDIS-21-0674RR Initial ×
mail.google.com/mail/u/0/?ik=94e8bbcc0f&view=pt&search=all&permthid=thread-f%3A1721296325383708665&dsqt=1&simpl=msg-f%3A1721296325383708665&si...
Aplicaciones   Primeros pasos   Últimas noticias   Importado desde Fi...   Medical Journals   Harvard University...   SJR : Scientific Jour...   LGALS1 galectin 1 [...   Lista de lectura

Pablo Carabias <carabiaspablo@gmail.com>
Para: María Fernanda Troncoso <ma.f.troncoso@gmail.com>

7 de enero de 2022, 12:31

Dear Fernanda,
I agree with the new authorships of our manuscript number: CDDIS-21-0674RR.

Best wishes

Pablo Carabias

El vie., 7 de enero de 2022 12:05, María Fernanda Troncoso <ma.f.troncoso@gmail.com> escribió:

Dear co-authors,

As requested by Cell Death and Disease, I would like to ask you to reply my email confirming that you agree with the new authorships of our manuscript:

Manuscript Number: CDDIS-21-0674RR

Title: Galectin-1 confers resistance to doxorubicin in hepatocellular carcinoma cells through modulation of P-glycoprotein expression

Authors: Pablo Carabias*, María Espelt*, María Bacigalupo, Paola Rojas, Luciana Sarrias, Ayelén Rubin, Nicolás Saffioti, María Elola, Juan Rossi, Carlota Wolfenstein-Todel, Gabriel Rabinovich, and María Troncoso

Pablo Carabias and María V Espelt contributed equally to this work
Luciana Sarrias was incorporated as co-author

Thanks!

Best wishes
Fernanda

---------- Forwarded message ---------
De: <cddisease@springernature.com>
Date: vie, 7 ene 2022 a las 8:48
Subject: CDDIS-21-0674RR Initial Quality Check

06:26 p.m.
07/01/2022

CDDIS-21-0674RR Initial Quality | Gmail - CDDIS-21-0674RR Initial

mail.google.com/mail/u/0/?ik=94e8bbcc0f&view=pt&search=all&permthid=thread-f%3A1721296325383708665&dsqt=1&simpl=msg-f%3A1721296325383708665&si...

Aplicaciones | Primeros pasos | Últimas noticias | Importado desde Fi... | Medical Journals | Harvard University... | SJR : Scientific Jour... | LGALS1 galectin 1 [... | Lista de lectura

Maria Victoria <vicespelt@gmail.com>
Para: María Fernanda Troncoso <ma.f.troncoso@gmail.com>

7 de enero de 2022, 10:17

Dear Fernanda,
I agree with the new authorships of the manuscript

Manuscript Number: CDDIS-21-0674RR

Title: Galectin-1 confers resistance to doxorubicin in hepatocellular carcinoma cells through modulation of P-glycoprotein expression
Authors: Pablo Carabias*, María Espelt*, María Bacigalupo, Paola Rojas, Luciana Sarrias, Ayelén Rubin, Nicolás Saffioti, María Elola, Juan Rossi, Carlota Wolfenstein-Todel, Gabriel Rabinovich, and María Troncoso

Regards,

Dr. Espelt

El vie, 7 ene 2022 a las 12:05, María Fernanda Troncoso (<ma.f.troncoso@gmail.com>) escribió:

> Dear co-authors,
>
> As requested by Cell Death and Disease, I would like to ask you to reply my email confirming that you agree with the new authorships of our manuscript:
>
> **Manuscript Number: CDDIS-21-0674RR**
>
> **Title: Galectin-1 confers resistance to doxorubicin in hepatocellular carcinoma cells through modulation of P-glycoprotein expression**
>
> **Authors: Pablo Carabias*, María Espelt*, María Bacigalupo, Paola Rojas, Luciana Sarrias, Ayelén Rubin, Nicolás Saffioti, María Elola, Juan Rossi, Carlota Wolfenstein-Todel, Gabriel Rabinovich, and María Troncoso**
>
> **Pablo Carabias and María V Espelt contributed equally to this work**
> **Luciana Sarrias was incorporated as co-author**
>
> Thanks!
>
> Best wishes
> Fernanda

06:26 p.m.
07/01/2022

CDDIS-21-0674RR Initial Quality × Gmail - CDDIS-21-0674RR Initial ×
mail.google.com/mail/u/0/?ik=94e8bbcc0f&view=pt&search=all&permthid=thread-f%3A1721296325383708665&dsqt=1&simpl=msg-f%3A1721296325383708665&si...
Aplicaciones Primeros pasos Últimas noticias Importado desde Fi... Medical Journals Harvard University... SJR : Scientific Jour... LGALS1 galectin 1 [... Lista de lectura

fernanda@qb.ffyb.uba.ar

María Lorena Bacigalupo <lorebaci@gmail.com> 7 de enero de 2022, 12:12
Para: María Fernanda Troncoso <ma.f.troncoso@gmail.com>

Dear Fernanda,

I agree with the new authorships of our manuscript:
Manuscript Number: CDDIS-21-0674RR

Best
María Lorena Bacigalupo

El vie., 7 de enero de 2022 12:05, María Fernanda Troncoso <ma.f.troncoso@gmail.com> escribió:

> Dear co-authors,
>
> As requested by Cell Death and Disease, I would like to ask you to reply my email confirming that you agree with the new authorships of our manuscript:
>
> **Manuscript Number: CDDIS-21-0674RR**
>
> **Title: Galectin-1 confers resistance to doxorubicin in hepatocellular carcinoma cells through modulation of P-glycoprotein expression**
>
> **Authors: Pablo Carabias*, María Espelt*, María Bacigalupo, Paola Rojas, Luciana Sarrias, Ayelén Rubin, Nicolás Saffioti, María Elola, Juan Rossi, Carlota Wolfenstein-Todel, Gabriel Rabinovich, and María Troncoso**
>
> **Pablo Carabias and María V Espelt contributed equally to this work**
> **Luciana Sarrias was incorporated as co-author**
>
> Thanks!
>
> Best wishes
> Fernanda
>
> ---------- Forwarded message ---------
> De: <cddisease@springernature.com>

06:22 p.m.
07/01/2022

CDDIS-21-0674RR Initial Quality ✕ Gmail - CDDIS-21-0674RR Initial ✕ +

mail.google.com/mail/u/0/?ik=94e8bbcc0f&view=pt&search=all&permthid=thread-f%3A1721296325383708665&dsqt=1&simpl=msg-f%3A1721296325383708665&si...

Aplicaciones Primeros pasos Últimas noticias Importado desde Fi... Medical Journals Harvard University... SJR : Scientific Jour... LGALS1 galectin 1 [... » Lista de lectura

Paola Rojas <parojas2010@gmail.com> 7 de enero de 2022, 13:00
Para: María Fernanda Troncoso <ma.f.troncoso@gmail.com>

Hi Fernanda, yes I agree.

Best
Paola Rojas

El vie, 7 ene 2022 a las 12:05, María Fernanda Troncoso (<ma.f.troncoso@gmail.com>) escribió:

Dear co-authors,

As requested by Cell Death and Disease, I would like to ask you to reply my email confirming that you agree with the new authorships of our manuscript:

Manuscript Number: CDDIS-21-0674RR

Title: Galectin-1 confers resistance to doxorubicin in hepatocellular carcinoma cells through modulation of P-glycoprotein expression

Authors: Pablo Carabias*, María Espelt*, María Bacigalupo, Paola Rojas, Luciana Sarrias, Ayelén Rubin, Nicolás Saffioti, María Elola, Juan Rossi, Carlota Wolfenstein-Todel, Gabriel Rabinovich, and María Troncoso

Pablo Carabias and María V Espelt contributed equally to this work
Luciana Sarrias was incorporated as co-author

Thanks!

Best wishes
Fernanda

---------- Forwarded message ---------
De: <cddisease@springernature.com>
Date: vie, 7 ene 2022 a las 8:48
Subject: CDDIS-21-0674RR Initial Quality Check
To: <ma.f.troncoso@gmail.com>

06:27 p.m.
07/01/2022

CDDIS-21-0674RR Initial Quality | Gmail - CDDIS-21-0674RR Initial

mail.google.com/mail/u/0/?ik=94e8bbcc0f&view=pt&search=all&permthid=thread-f%3A1721296325383708665&dsqt=1&simpl=msg-f%3A1721296325383708665&si...

Aplicaciones | Primeros pasos | Últimas noticias | Importado desde Fi... | Medical Journals | Harvard University... | SJR : Scientific Jour... | LGALS1 galectin 1 [... | Lista de lectura

Luciana Sarrias <lusarrias13@gmail.com> — 7 de enero de 2022, 12:31

Para: María Fernanda Troncoso <ma.f.troncoso@gmail.com>

Dear Dr María Fernanda, I agree with my addition in the authorship of the paper.

Best,
Luciana Sarrias

El vie, 7 ene 2022 a las 12:05, María Fernanda Troncoso (<ma.f.troncoso@gmail.com>) escribió:

Dear co-authors,

As requested by Cell Death and Disease, I would like to ask you to reply my email confirming that you agree with the new authorships of our manuscript:

Manuscript Number: CDDIS-21-0674RR

Title: Galectin-1 confers resistance to doxorubicin in hepatocellular carcinoma cells through modulation of P-glycoprotein expression

Authors: Pablo Carabias*, María Espelt*, María Bacigalupo, Paola Rojas, Luciana Sarrias, Ayelén Rubin, Nicolás Saffioti, María Elola, Juan Rossi, Carlota Wolfenstein-Todel, Gabriel Rabinovich, and María Troncoso

Pablo Carabias and María V Espelt contributed equally to this work
Luciana Sarrias was incorporated as co-author

Thanks!

Best wishes
Fernanda

---------- Forwarded message ---------
De: <cddisease@springernature.com>
Date: vie, 7 ene 2022 a las 8:48
Subject: CDDIS-21-0674RR Initial Quality Check
To: <ma.f.troncoso@gmail.com>

06:25 p.m.
07/01/2022

CDDIS-21-0674RR Initial Quality ✕ | Gmail - CDDIS-21-0674RR Initial ✕ | +

mail.google.com/mail/u/0/?ik=94e8bbcc0f&view=pt&search=all&permthid=thread-f%3A1721296325383708665&dsqt=1&simpl=msg-f%3A1721296325383708665&si...

Aplicaciones | Primeros pasos | Últimas noticias | Importado desde Fi... | Medical Journals | Harvard University... | SJR : Scientific Jour... | LGALS1 galectin 1 [... | » | Lista de lectura

Ayelen Rubin <aye.rubin@gmail.com> 7 de enero de 2022, 12:23
Para: María Fernanda Troncoso <ma.f.troncoso@gmail.com>

I agree to the authors changes.
Kind regards,

Ayelen Rubin

El vie., 7 de enero de 2022 12:05, María Fernanda Troncoso <ma.f.troncoso@gmail.com> escribió:

> Dear co-authors,
>
> As requested by Cell Death and Disease, I would like to ask you to reply my email confirming that you agree with the new authorships of our manuscript:
>
> **Manuscript Number: CDDIS-21-0674RR**
>
> **Title: Galectin-1 confers resistance to doxorubicin in hepatocellular carcinoma cells through modulation of P-glycoprotein expression**
>
> **Authors: Pablo Carabias*, María Espelt*, María Bacigalupo, Paola Rojas, Luciana Sarrias, Ayelén Rubin, Nicolás Saffioti, María Elola, Juan Rossi, Carlota Wolfenstein-Todel, Gabriel Rabinovich, and María Troncoso**
>
> **Pablo Carabias and María V Espelt contributed equally to this work**
> **Luciana Sarrias was incorporated as co-author**
>
> Thanks!
>
> Best wishes
> Fernanda
>
> ---------- Forwarded message ---------
> De: <cddisease@springernature.com>
> Date: vie, 7 ene 2022 a las 8:48
> Subject: CDDIS-21-0674RR Initial Quality Check
> To: <ma.f.troncoso@gmail.com>

06:25 p.m.
07/01/2022

CDDIS-21-0674RR Initial Quality ✕ | Gmail - CDDIS-21-0674RR Initial ✕ | +

mail.google.com/mail/u/0/?ik=94e8bbcc0f&view=pt&search=all&permthid=thread-f%3A1721296325383708665&dsqt=1&simpl=msg-f%3A1721296325383708665&si...

Aplicaciones | Primeros pasos | Últimas noticias | Importado desde Fi... | Medical Journals | Harvard University... | SJR : Scientific Jour... | LGALS1 galectin 1 [... | » | Lista de lectura

Nicolas Andres Saffioti <nsaffioti@gmail.com> — 7 de enero de 2022, 12:28
Para: María Fernanda Troncoso <ma.f.troncoso@gmail.com>

Dear Fernanda,

I agree with the new authorship of the paper.

Best regards

El vie, 7 ene 2022 a las 12:05, María Fernanda Troncoso (<ma.f.troncoso@gmail.com>) escribió:

> Dear co-authors,
>
> As requested by Cell Death and Disease, I would like to ask you to reply my email confirming that you agree with the new authorships of our manuscript:
>
> **Manuscript Number: CDDIS-21-0674RR**
>
> **Title: Galectin-1 confers resistance to doxorubicin in hepatocellular carcinoma cells through modulation of P-glycoprotein expression**
>
> **Authors: Pablo Carabias*, María Espelt*, María Bacigalupo, Paola Rojas, Luciana Sarrias, Ayelén Rubin, Nicolás Saffioti, María Elola, Juan Rossi, Carlota Wolfenstein-Todel, Gabriel Rabinovich, and María Troncoso**
>
> **Pablo Carabias and María V Espelt contributed equally to this work**
> **Luciana Sarrias was incorporated as co-author**
>
> Thanks!
>
> Best wishes
> Fernanda
>
> ---------- Forwarded message ---------
> De: <cddisease@springernature.com>
> Date: vie, 7 ene 2022 a las 8:48
> Subject: CDDIS-21-0674RR Initial Quality Check
> To: <ma.f.troncoso@gmail.com>

06:25 p.m.
07/01/2022

CDDIS-21-0674RR Initial Quality | Gmail - CDDIS-21-0674RR Initial

mail.google.com/mail/u/0/?ik=94e8bbcc0f&view=pt&search=all&permthid=thread-f%3A1721296325383708665&dsqt=1&simpl=msg-f%3A1721296325383708665&si...

Aplicaciones | Primeros pasos | Últimas noticias | Importado desde Fi... | Medical Journals | Harvard University... | SJR : Scientific Jour... | LGALS1 galectin 1 [... | Lista de lectura

maria teresa elola <mt_elola@yahoo.com> 7 de enero de 2022, 12:21
Para: María Fernanda Troncoso <ma.f.troncoso@gmail.com>

Dear Dr. María F. Troncoso,

I agree with the modified list of authors for the Manuscript Number CDDIS-21-0674RR entitled
"Galectin-1 confers resistance to doxorubicin in hepatocellular carcinoma cells through modulation of P-glycoprotein expression".

Thus, the new authors are as follows:
Pablo Carabias*, María Espelt*, María Bacigalupo, Paola Rojas, Luciana Sarrias, Ayelén Rubin, Nicolás Saffioti, María Elola, Juan Rossi, Carlota Wolfenstein-Todel, Gabriel Rabinovich, and María Troncoso.

It should be pointed out that Pablo Carabias and María Espelt share the first authorship.

Yours sincerely,
Best regards,

María T. Elola.

María T. Elola. PhD.
Institute of Biological Chemistry and Biophysics (UBA-CONICET)
Department of Biological Chemistry, School of Pharmacy and Biochemistry
University of Buenos Aires, Junín 956, C1113AAD
Buenos Aires, Argentina
(5411) 5287 4122.

06:24 p.m.
07/01/2022

CDDIS-21-0674RR Initial Quality × | Gmail - CDDIS-21-0674RR Initial ×

mail.google.com/mail/u/0/?ik=94e8bbcc0f&view=pt&search=all&permthid=thread-f%3A1721296325383708665&dsqt=1&simpl=msg-f%3A1721296325383708665&si...

Aplicaciones | Primeros pasos | Últimas noticias | Importado desde Fi... | Medical Journals | Harvard University... | SJR : Scientific Jour... | LGALS1 galectin 1 [... | » | Lista de lectura

fernanda@qb.ffyb.uba.ar

Juan Pablo Rossi <vonckers@gmail.com> 7 de enero de 2022, 12:14
Para: María Fernanda Troncoso <ma.f.troncoso@gmail.com>

I agree with the authors' modifications

Regards
Juan Pablo F.C. Rossi

Libre de virus. www.avast.com

El vie, 7 ene 2022 a las 12:05, María Fernanda Troncoso (<ma.f.troncoso@gmail.com>) escribió:

Dear co-authors,

As requested by Cell Death and Disease, I would like to ask you to reply my email confirming that you agree with the new authorships of our manuscript:

Manuscript Number: CDDIS-21-0674RR

Title: Galectin-1 confers resistance to doxorubicin in hepatocellular carcinoma cells through modulation of P-glycoprotein expression

Authors: Pablo Carabias*, María Espelt*, María Bacigalupo, Paola Rojas, Luciana Sarrias, Ayelén Rubin, Nicolás Saffioti, María Elola, Juan Rossi, Carlota Wolfenstein-Todel, Gabriel Rabinovich, and María Troncoso

Pablo Carabias and María V Espelt contributed equally to this work
Luciana Sarrias was incorporated as co-author

Thanks!

Best wishes
Fernanda

06:23 p.m.
07/01/2022

CDDIS-21-0674RR Initial Quality | Gmail - CDDIS-21-0674RR Initial

mail.google.com/mail/u/0/?ik=94e8bbcc0f&view=pt&search=all&permthid=thread-f%3A1721296325383708665&dsqt=1&simpl=msg-f%3A1721296325383708665&si...

Aplicaciones | Primeros pasos | Últimas noticias | Importado desde Fi... | Medical Journals | Harvard University... | SJR : Scientific Jour... | LGALS1 galectin 1 [... | Lista de lectura

Carlota Wolfenstein de Todel <cwtodel2002@yahoo.com.ar> — 7 de enero de 2022, 12:59
Para: María Fernanda Troncoso <ma.f.troncoso@gmail.com>

Dear Fernanda:
I agree
Best wishes
Carlota

Carlota Wolfenstein-Todel

El viernes, 7 de enero de 2022 12:05:48 ART, María Fernanda Troncoso <ma.f.troncoso@gmail.com> escribió:

Dear co-authors,

As requested by Cell Death and Disease, I would like to ask you to reply my email confirming that you agree with the new authorships of our manuscript:

Manuscript Number: CDDIS-21-0674RR

Title: Galectin-1 confers resistance to doxorubicin in hepatocellular carcinoma cells through modulation of P-glycoprotein expression

Authors: Pablo Carabias*, María Espelt*, María Bacigalupo, Paola Rojas, Luciana Sarrias, Ayelén Rubin, Nicolás Saffioti, María Elola, Juan Rossi, Carlota Wolfenstein-Todel, Gabriel Rabinovich, and María Troncoso

Pablo Carabias and María V Espelt contributed equally to this work
Luciana Sarrias was incorporated as co-author

Thanks!

Best wishes
Fernanda

06:27 p.m.
07/01/2022

CDDIS-21-0674RR Initial Quality
Gmail - CDDIS-21-0674RR Initial
mail.google.com/mail/u/0/?ik=94e8bbcc0f&view=pt&search=all&permthid=thread-f%3A1721296325383708665&dsqt=1&simpl=msg-f%3A1721296325383708665&si...
Aplicaciones Primeros pasos Últimas noticias Importado desde Fi... Medical Journals Harvard University... SJR : Scientific Jour... LGALS1 galectin 1 [... Lista de lectura

Gabriel A. Rabinovich <gabyrabi@gmail.com> 7 de enero de 2022, 13:14
Para: María Fernanda Troncoso <ma.f.troncoso@gmail.com>
Cc: Pablo Carabias <carabiaspablo@gmail.com>, Maria Victoria Espelt <vicespelt@gmail.com>, María Lorena Bacigalupo <lorebaci@gmail.com>, Paola Rojas <parojas2010@gmail.com>, Luciana Sarrias <lusarrias13@gmail.com>, aye.rubin@gmail.com, Nicolas Andres Saffioti <nsaffioti@gmail.com>, maria teresa elola <mt_elola@yahoo.com>, Jean Paul Rossi <jprossi@qb.ffyb.uba.ar>, Vonckers <vonckers@gmail.com>, Carlota Wolfenstein de Todel <cwtodel2002@yahoo.com.ar>

Dear Dr. Troncoso,

I fully agree with the new authorships of the manuscript CDDIS-21-0674RR

Best wishes,
Gabriel A. Rabinovich

El vie, 7 ene 2022 a las 12:05, María Fernanda Troncoso (<ma.f.troncoso@gmail.com>) escribió:

Dear co-authors,

As requested by Cell Death and Disease, I would like to ask you to reply my email confirming that you agree with the new authorships of our manuscript:

Manuscript Number: CDDIS-21-0674RR

Title: Galectin-1 confers resistance to doxorubicin in hepatocellular carcinoma cells through modulation of P-glycoprotein expression

Authors: Pablo Carabias*, María Espelt*, María Bacigalupo, Paola Rojas, Luciana Sarrias, Ayelén Rubin, Nicolás Saffioti, María Elola, Juan Rossi, Carlota Wolfenstein-Todel, Gabriel Rabinovich, and María Troncoso

Pablo Carabias and María V Espelt contributed equally to this work
Luciana Sarrias was incorporated as co-author

Thanks!

Best wishes
Fernanda

---------- Forwarded message ---------

06:28 p.m.
07/01/2022
